# Supplementary material for: Genomic basis of adaptation to serpentine soil in two Alyssum species shows convergence with Arabidopsis across 20 million years of divergence
Source: Ann Bot. 2025 Jul 10;138(3):851–67. doi: 10.1093/aob/mcaf141 (PMC13487438; doi:10.1093/aob/mcaf141)
Supplement: Supplementary file 1 [file mcaf141_supplementary_data.zip › Supplementary_Figures_2.pdf]

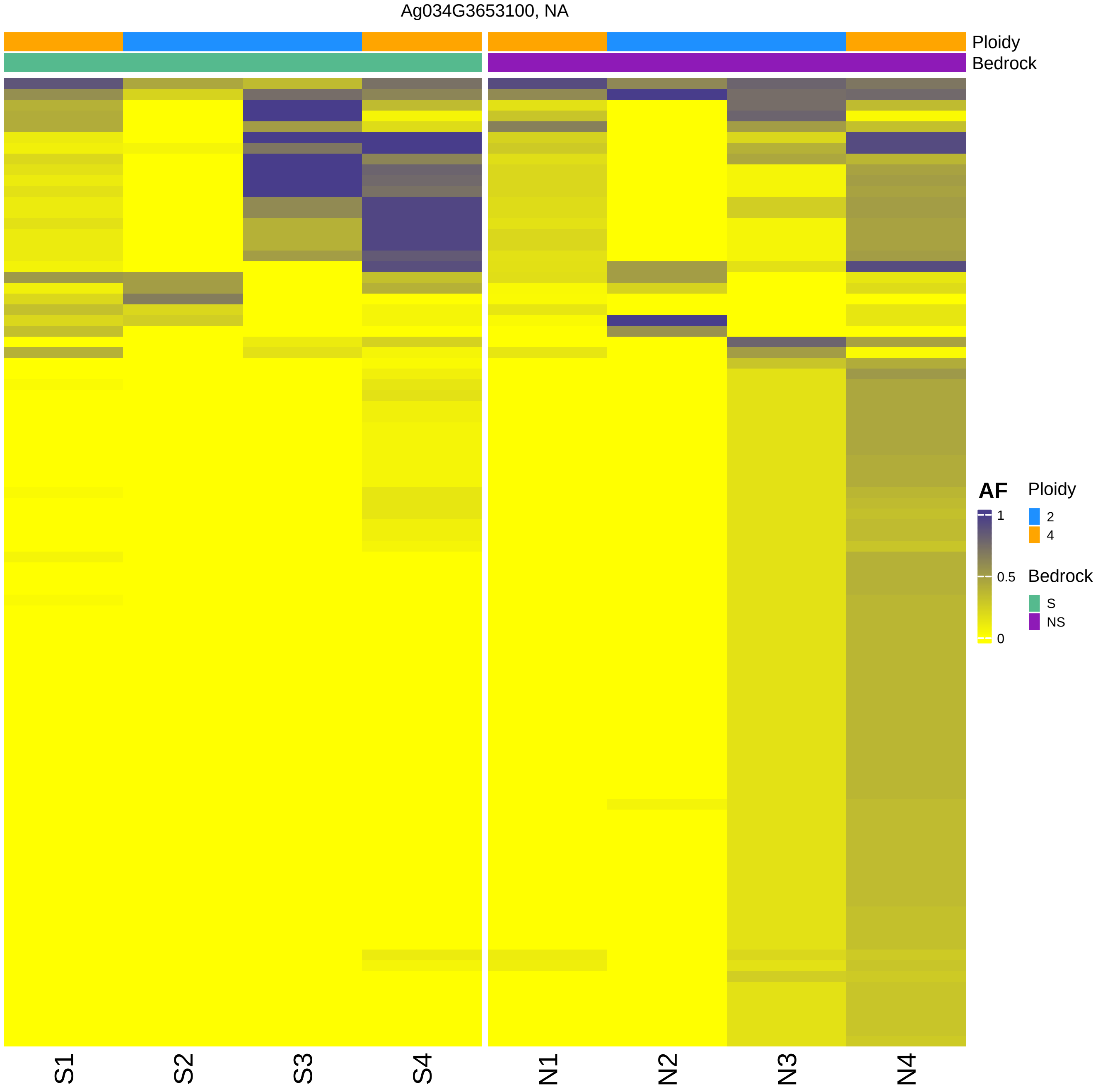

Ag034G3653100, NA
Ploidy
Bedrock
AF
1
0.5
0
Ploidy
2
4
Bedrock
S
NS
S1
S2
S3
S4
N1
N2
N3
N4

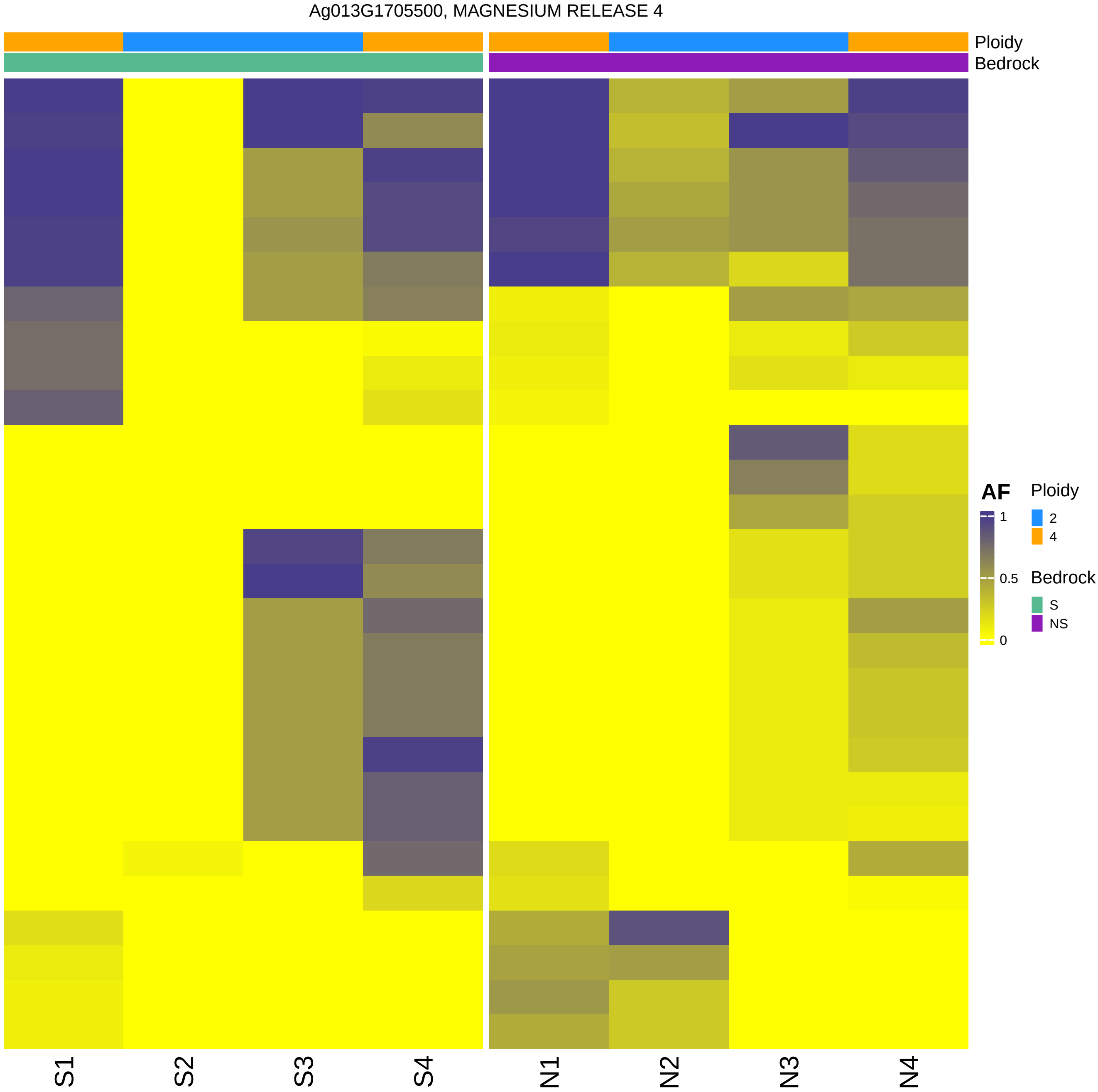

Ag013G1705500, MAGNESIUM RELEASE 4
Ploidy
Bedrock
AF
1
0.5
0
Ploidy
2
4
Bedrock
S
NS
S1
S2
S3
S4
N1
N2
N3
N4

g007G0896800, PEROXIREDOXIN IIC, thioredoxin−dependent peroxidase 2 (AT1G65970), PEROXIREDOXIN IID (AT1G60740)

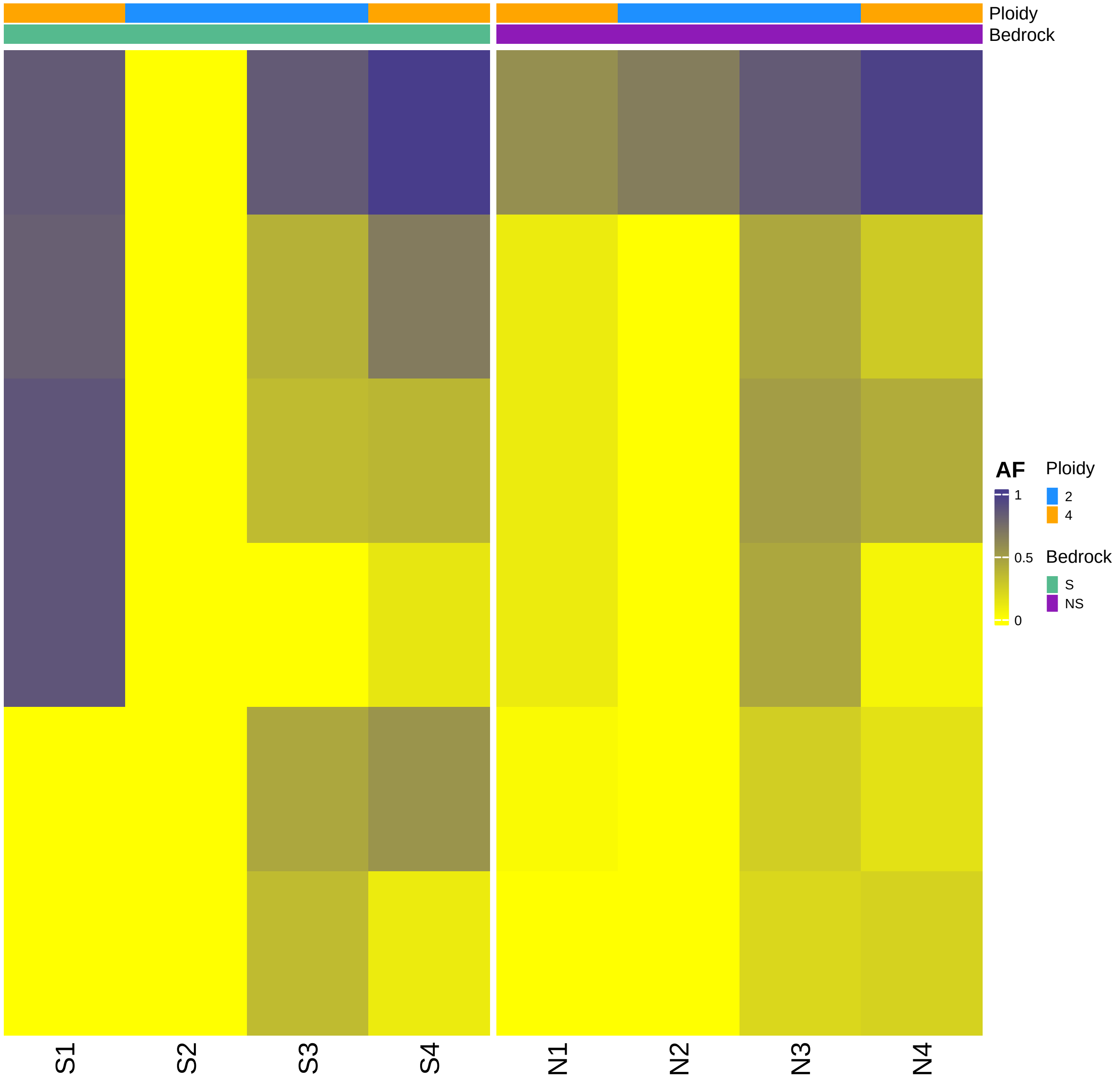

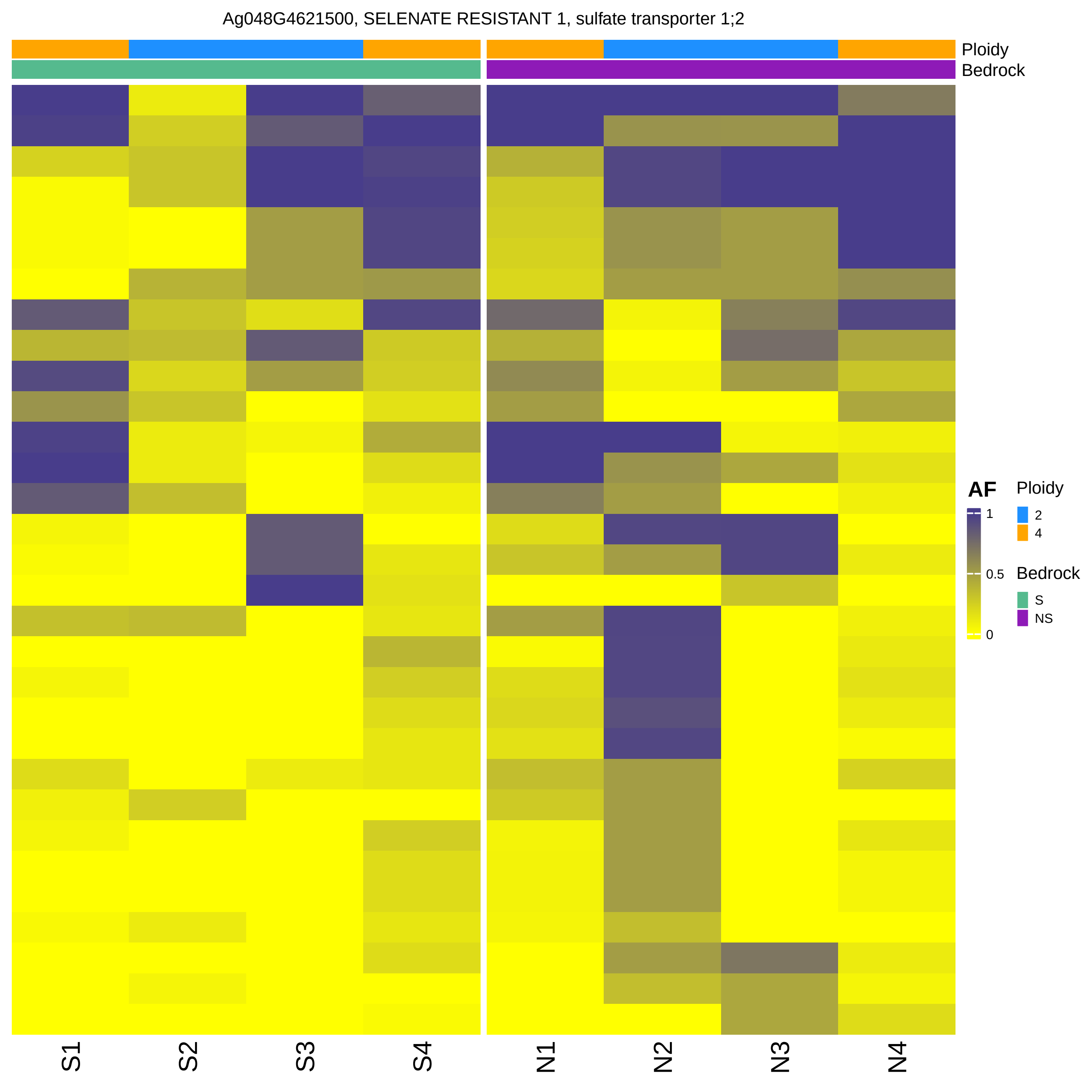

Ag048G4621500, SELENATE RESISTANT 1, sulfate transporter 1;2
Ploidy
Bedrock
AF
1
0.5
0
Ploidy
2
4
Bedrock
S
NS
S1
S2
S3
S4
N1
N2
N3
N4

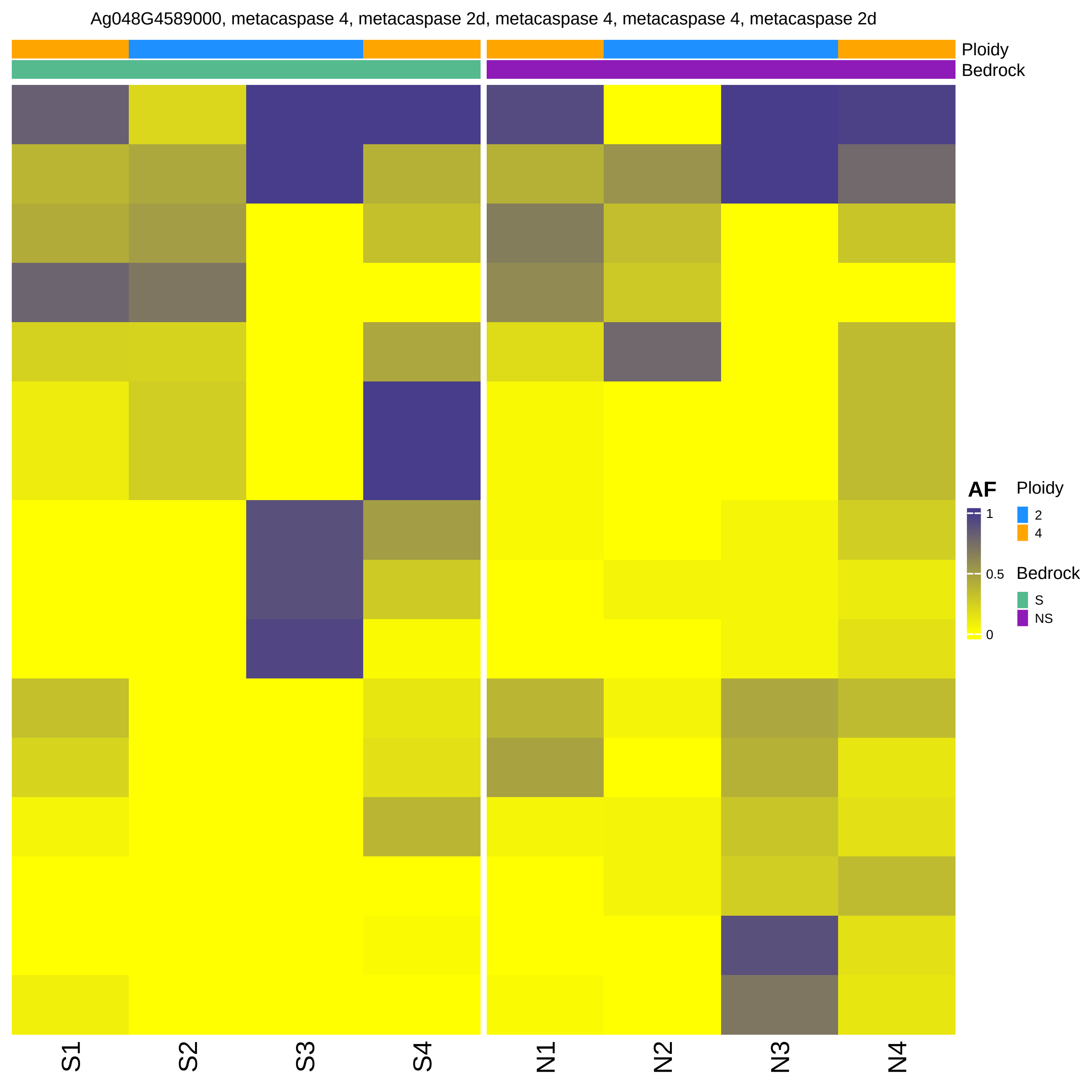
Ag048G4589000, metacaspase 4, metacaspase 2d, metacaspase 4, metacaspase 4, metacaspase 2d
Ploidy
Bedrock
AF
1
0.5
0
Ploidy
2
4
Bedrock
S
NS
S1
S2
S3
S4
N1
N2
N3
N4

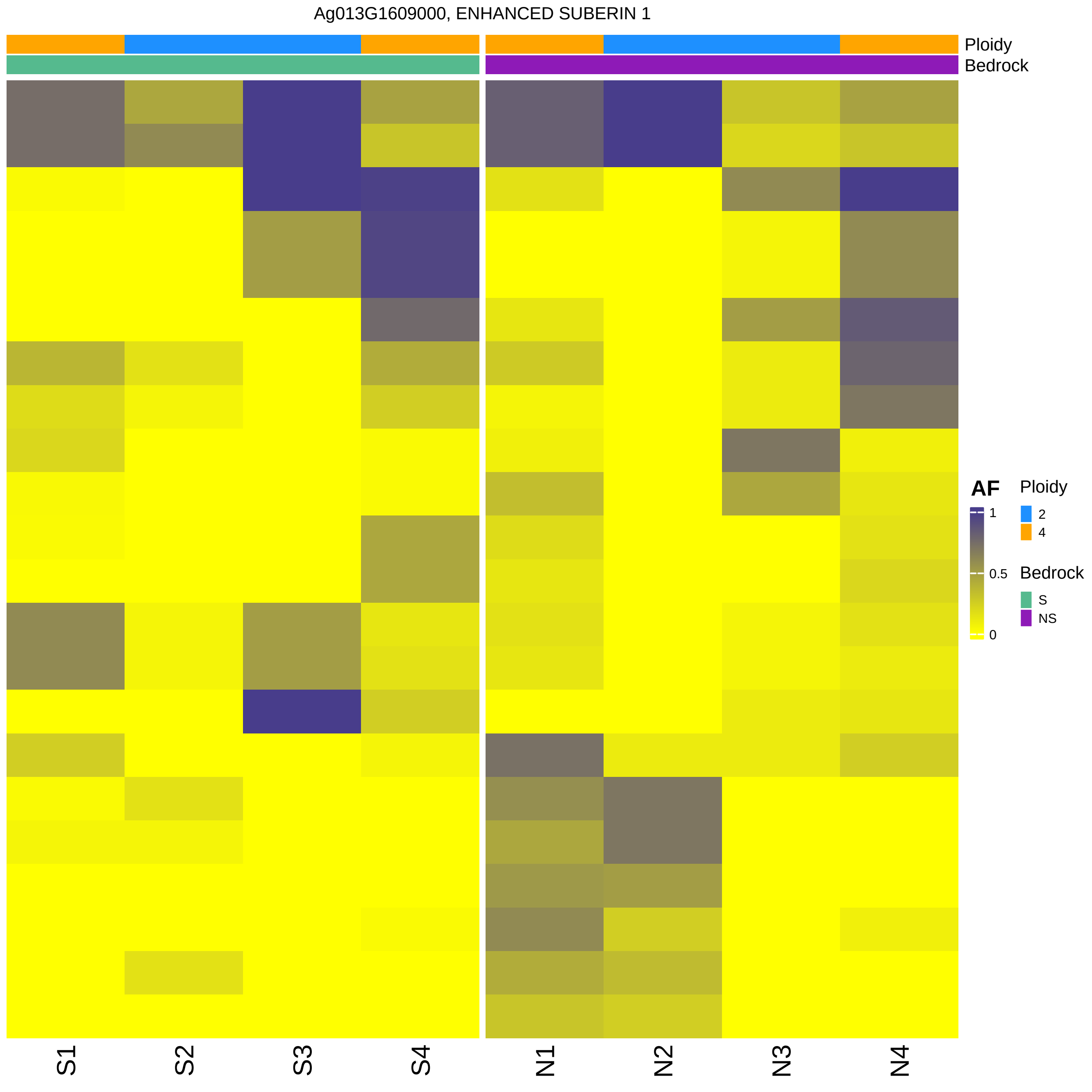
Ag013G1609000, ENHANCED SUBERIN 1
Ploidy
Bedrock
S1
S2
S3
S4
N1
N2
N3
N4
AF
1
0.5
0
Ploidy
2
4
Bedrock
S
NS

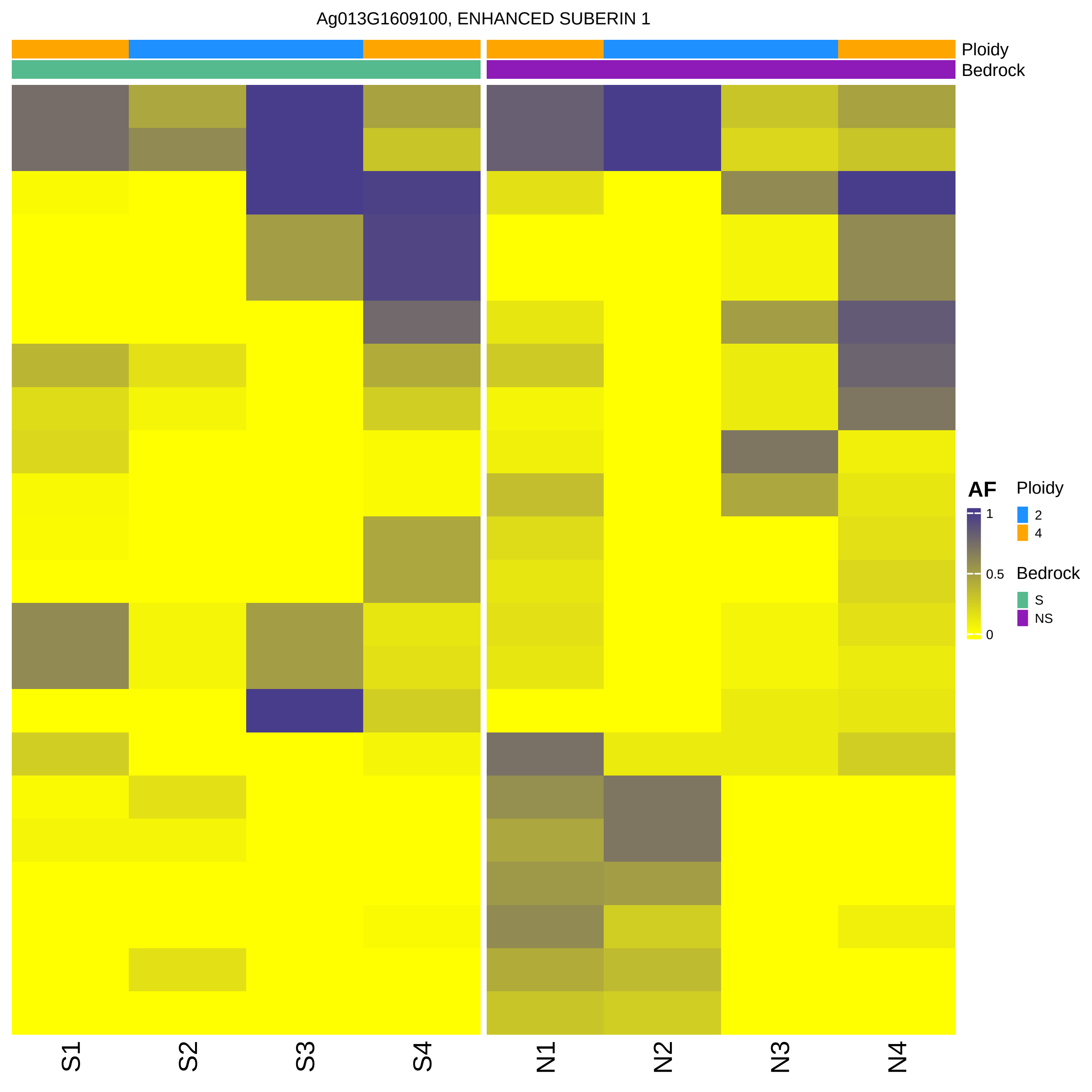

Ag013G1609100, ENHANCED SUBERIN 1
Ploidy
Bedrock
AF
1
0.5
0
Ploidy
2
4
Bedrock
S
NS
S1
S2
S3
S4
N1
N2
N3
N4

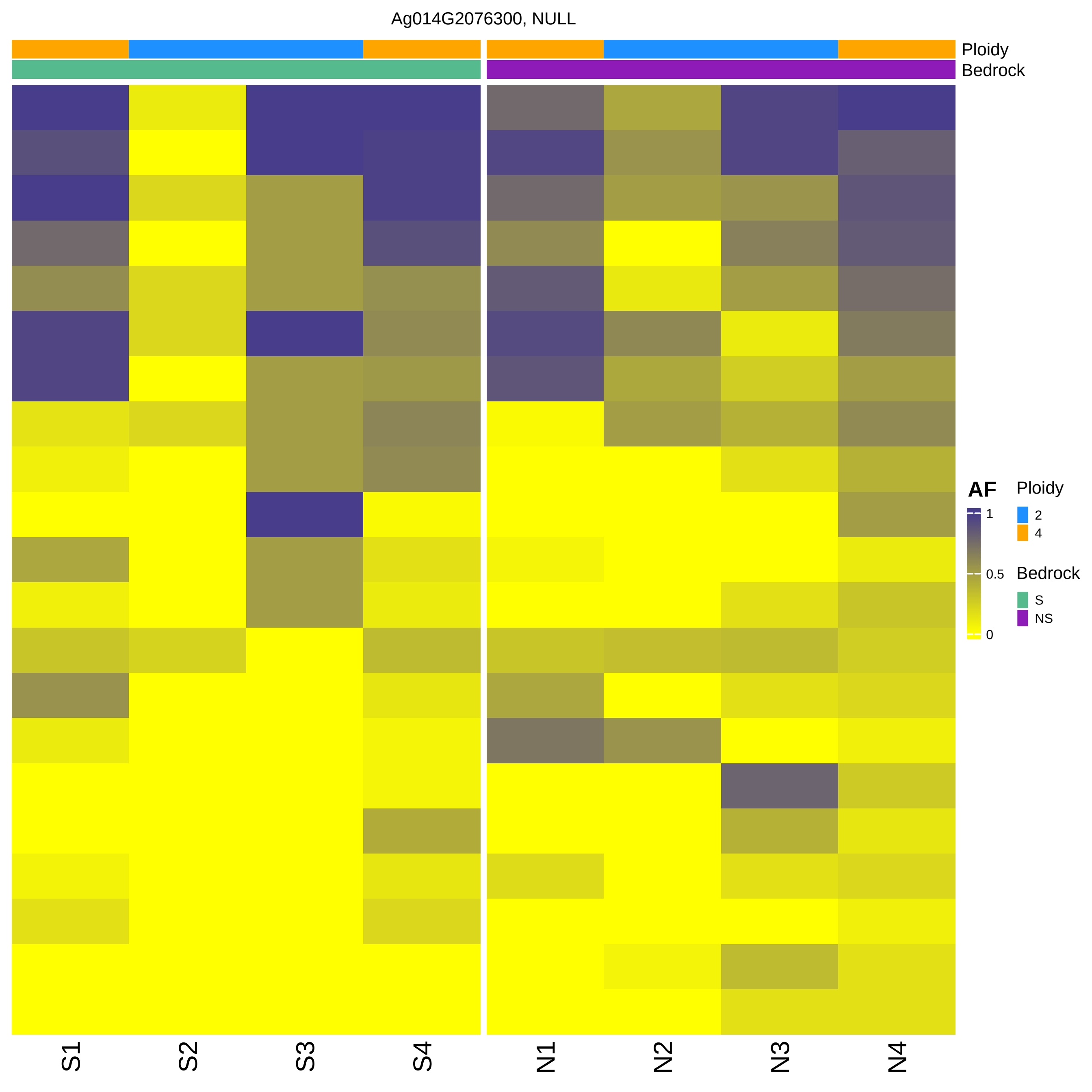

Ag014G2076300, NULL
Ploidy
Bedrock
AF
1
0.5
0
Ploidy
2
4
Bedrock
S
NS
S1
S2
S3
S4
N1
N2
N3
N4

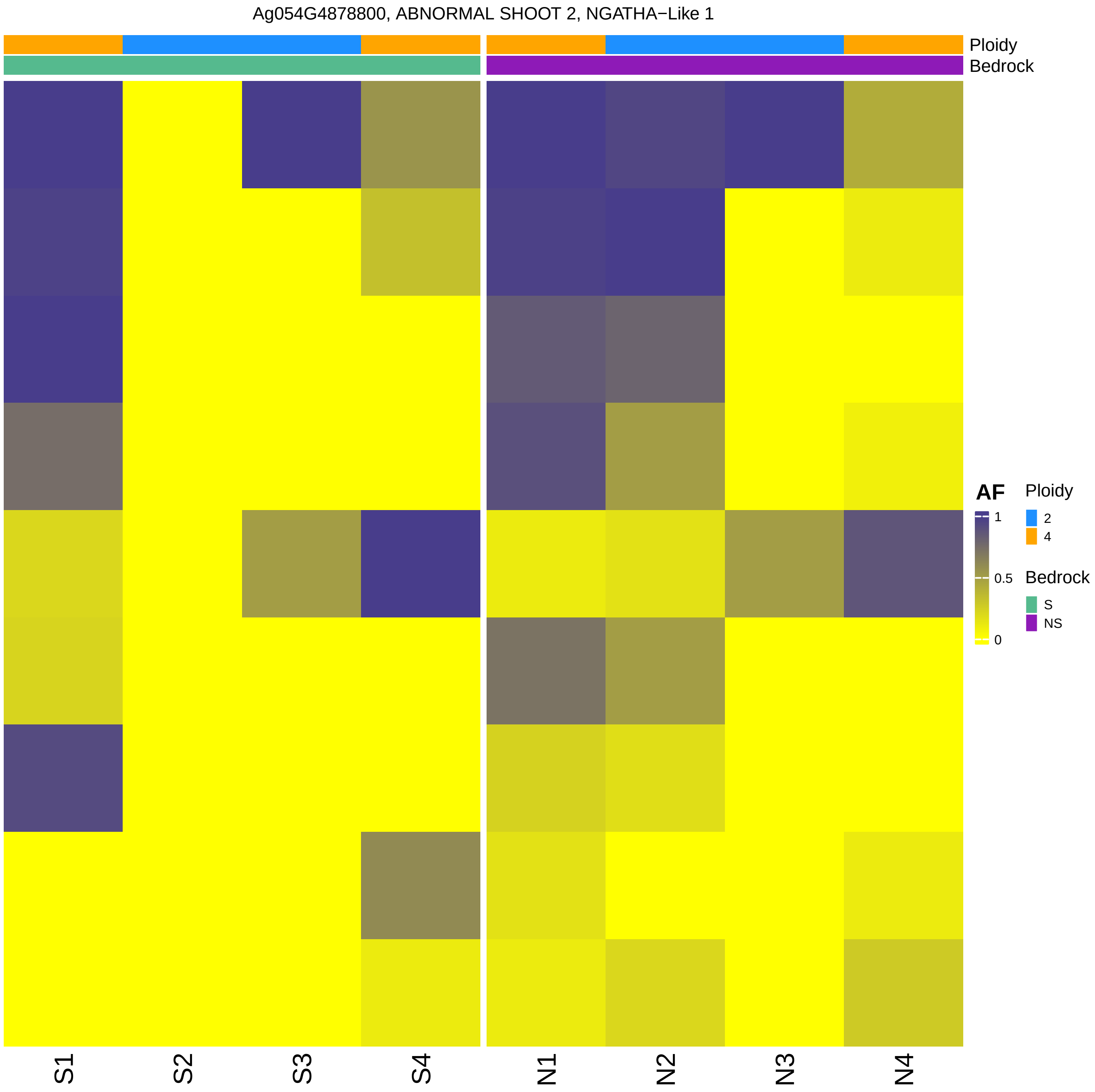
Ag054G4878800, ABNORMAL SHOOT 2, NGATHA−Like 1
Ploidy
Bedrock
AF
1
0.5
0
Ploidy
2
4
Bedrock
S
NS
S1
S2
S3
S4
N1
N2
N3
N4

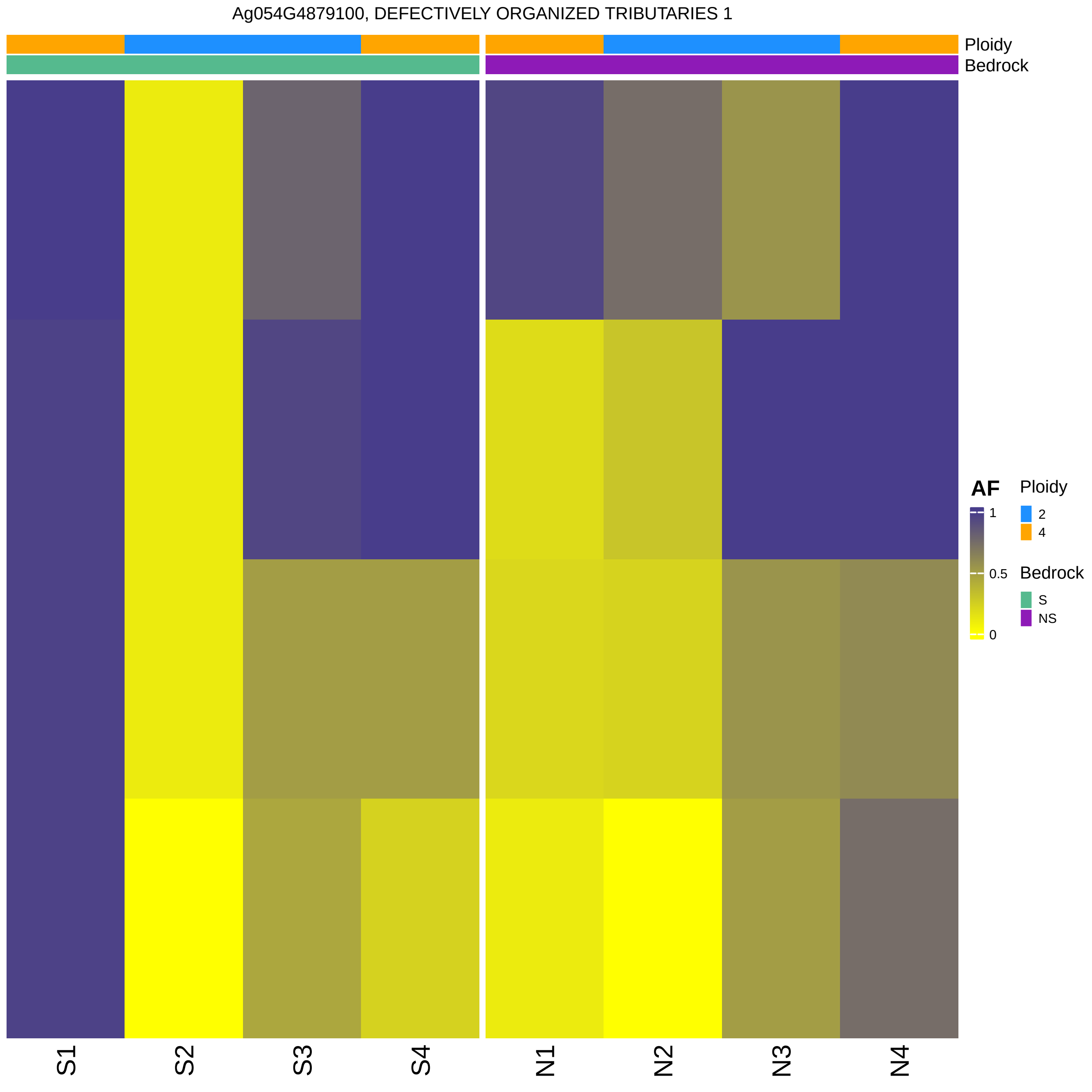

Ag054G4879100, DEFECTIVELY ORGANIZED TRIBUTARIES 1
Ploidy
Bedrock
AF
1
0.5
0
Ploidy
2
4
Bedrock
S
NS
S1
S2
S3
S4
N1
N2
N3
N4

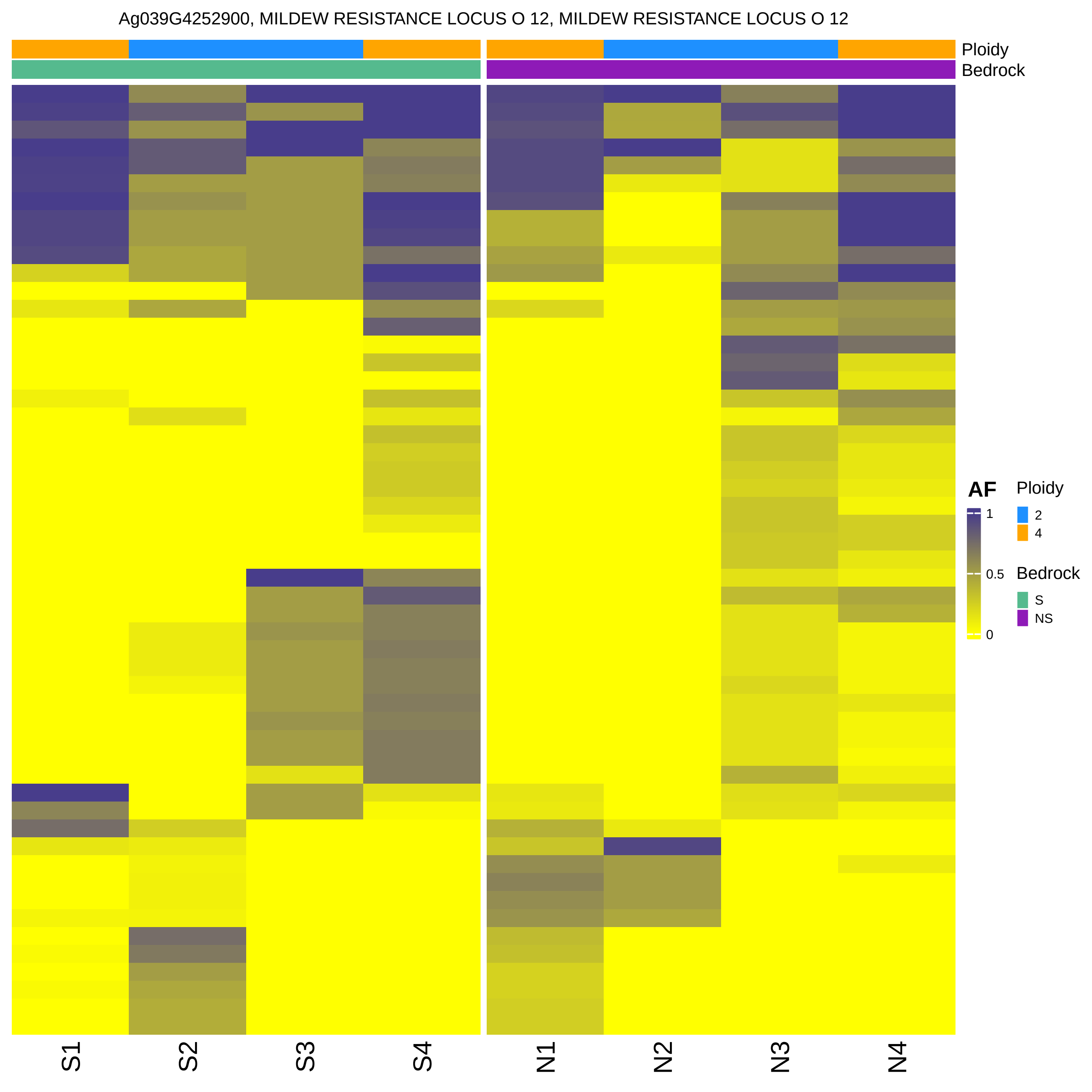
Ag039G4252900, MILDEW RESISTANCE LOCUS O 12, MILDEW RESISTANCE LOCUS O 12
Ploidy
Bedrock
AF
1
0.5
0
Ploidy
2
4
Bedrock
S
NS
S1
S2
S3
S4
N1
N2
N3
N4

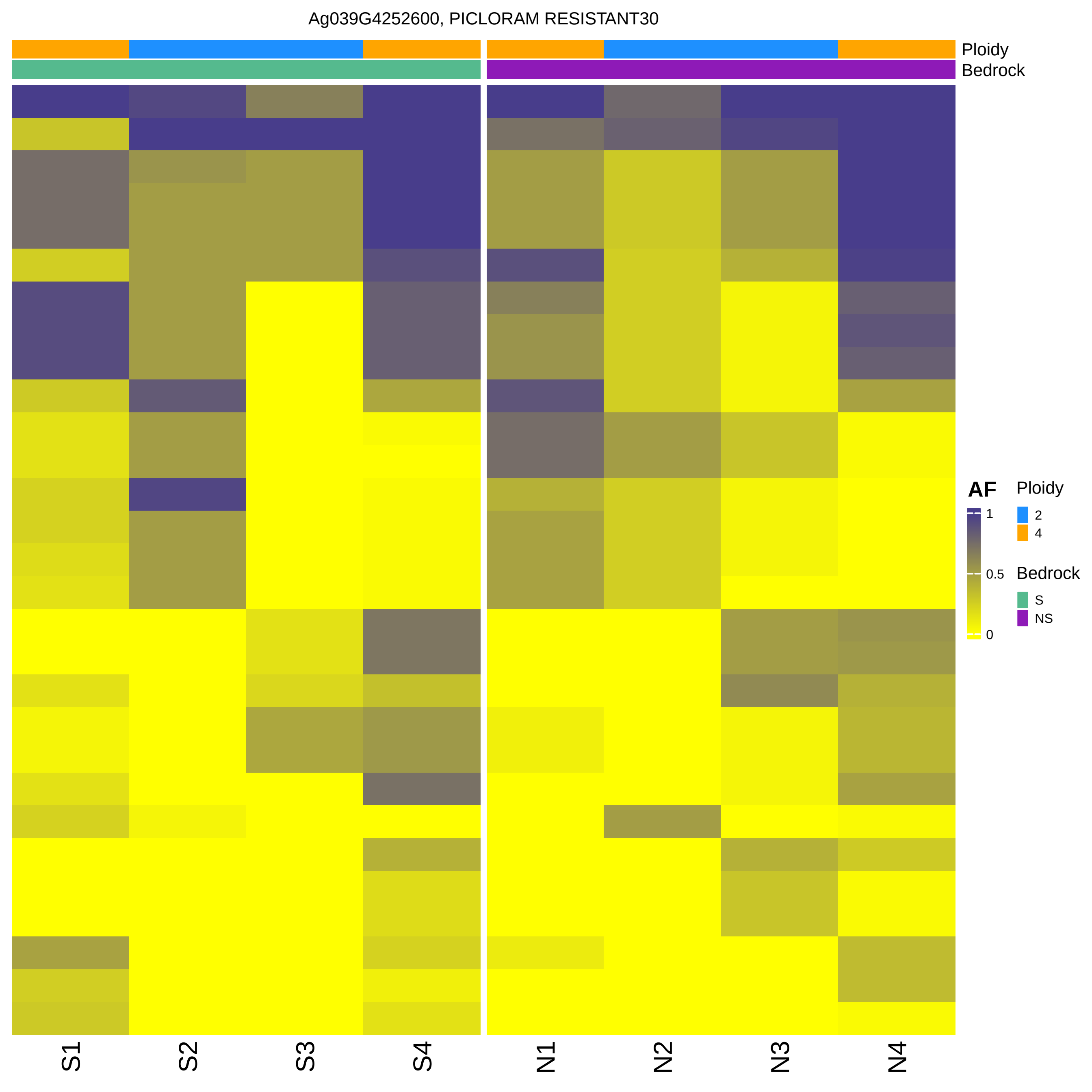
Ag039G4252600, PICLORAM RESISTANT30
Ploidy
Bedrock
AF
1
0.5
0
Ploidy
2
4
Bedrock
S
NS
S1
S2
S3
S4
N1
N2
N3
N4

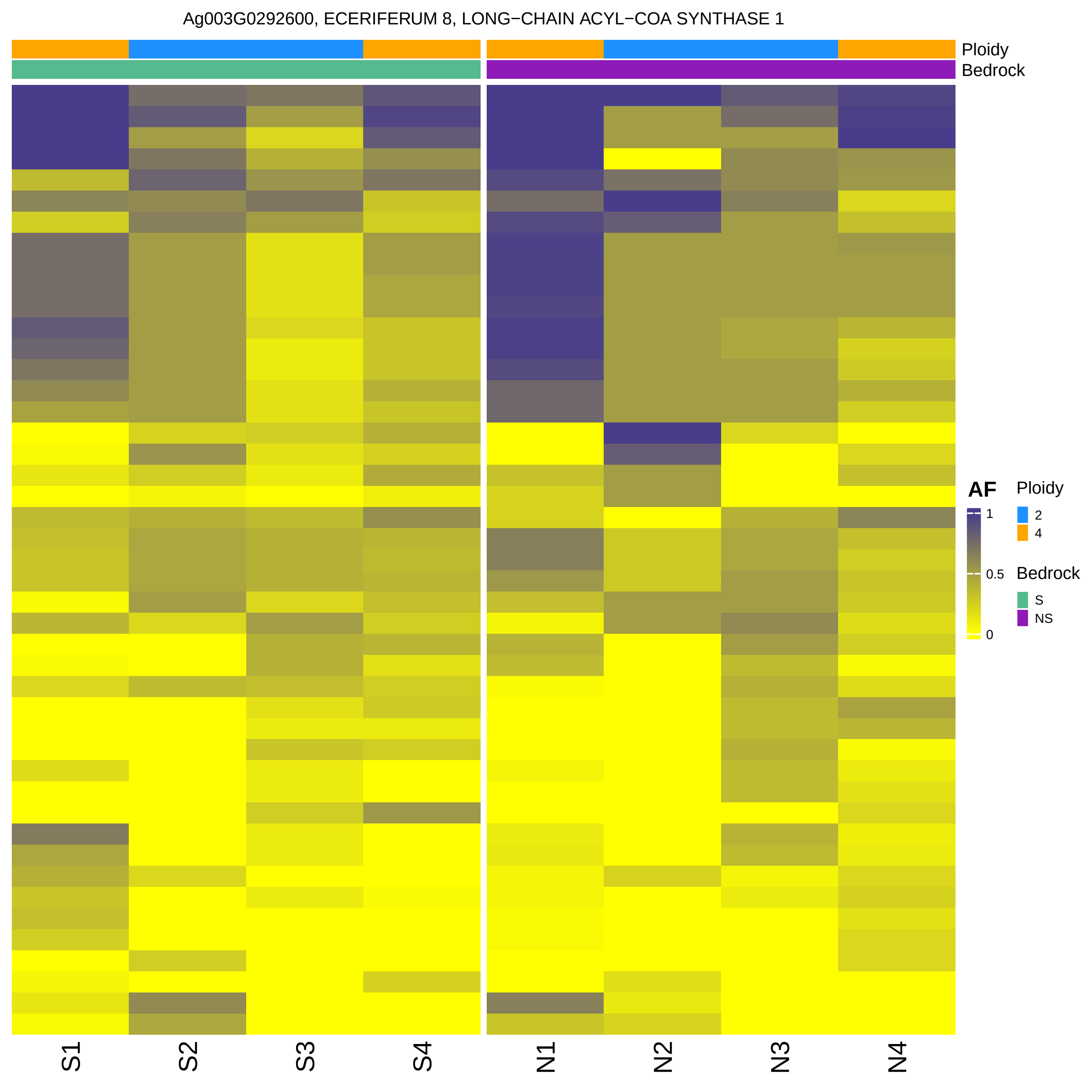

Ag003G0292600, ECERIFERUM 8, LONG−CHAIN ACYL−COA SYNTHASE 1
Ploidy
Bedrock
AF
1
0.5
0
Ploidy
2
4
Bedrock
S
NS
S1
S2
S3
S4
N1
N2
N3
N4

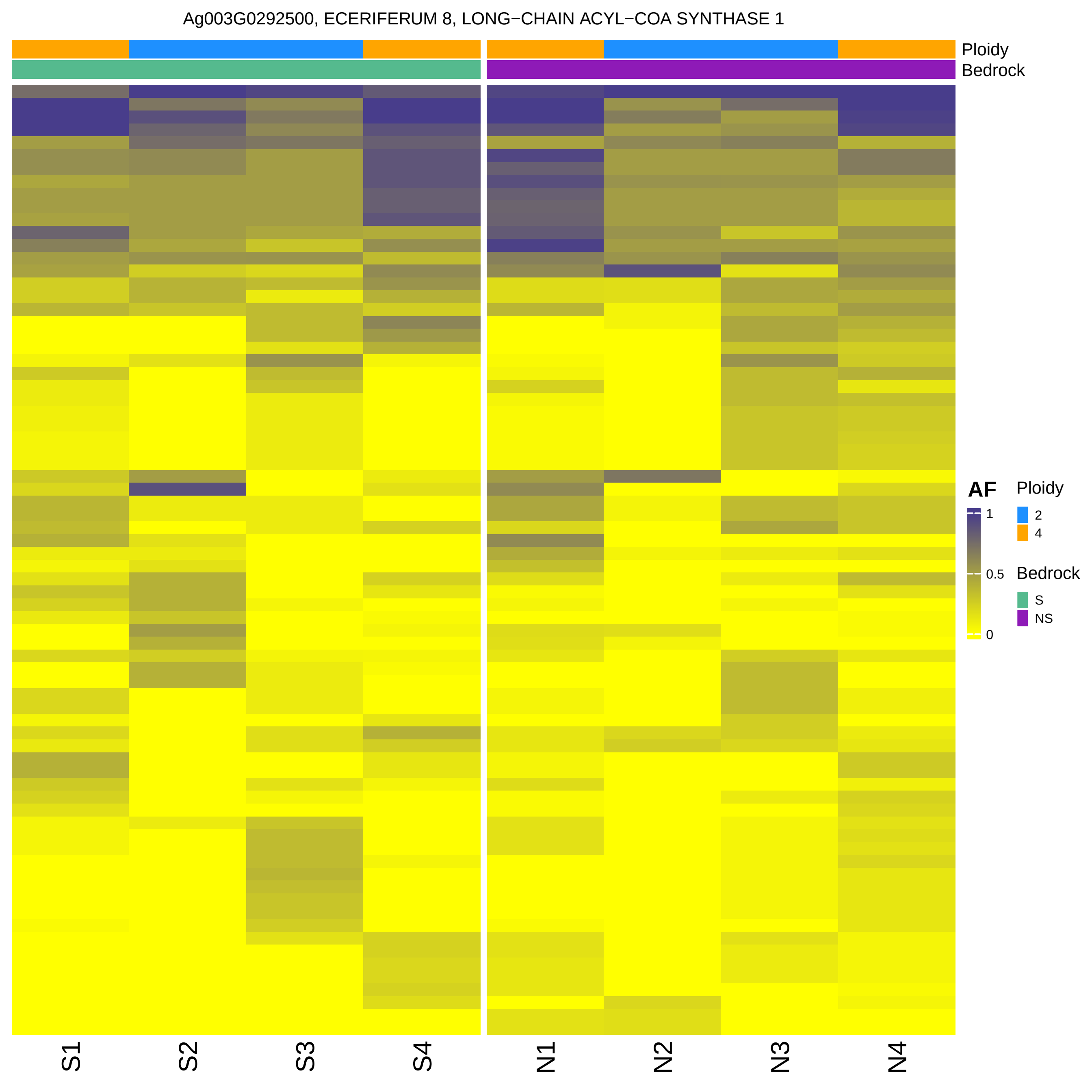
Ag003G0292500, ECERIFERUM 8, LONG−CHAIN ACYL−COA SYNTHASE 1
Ploidy
Bedrock
AF
1
0.5
0
Ploidy
2
4
Bedrock
S
NS
S1
S2
S3
S4
N1
N2
N3
N4

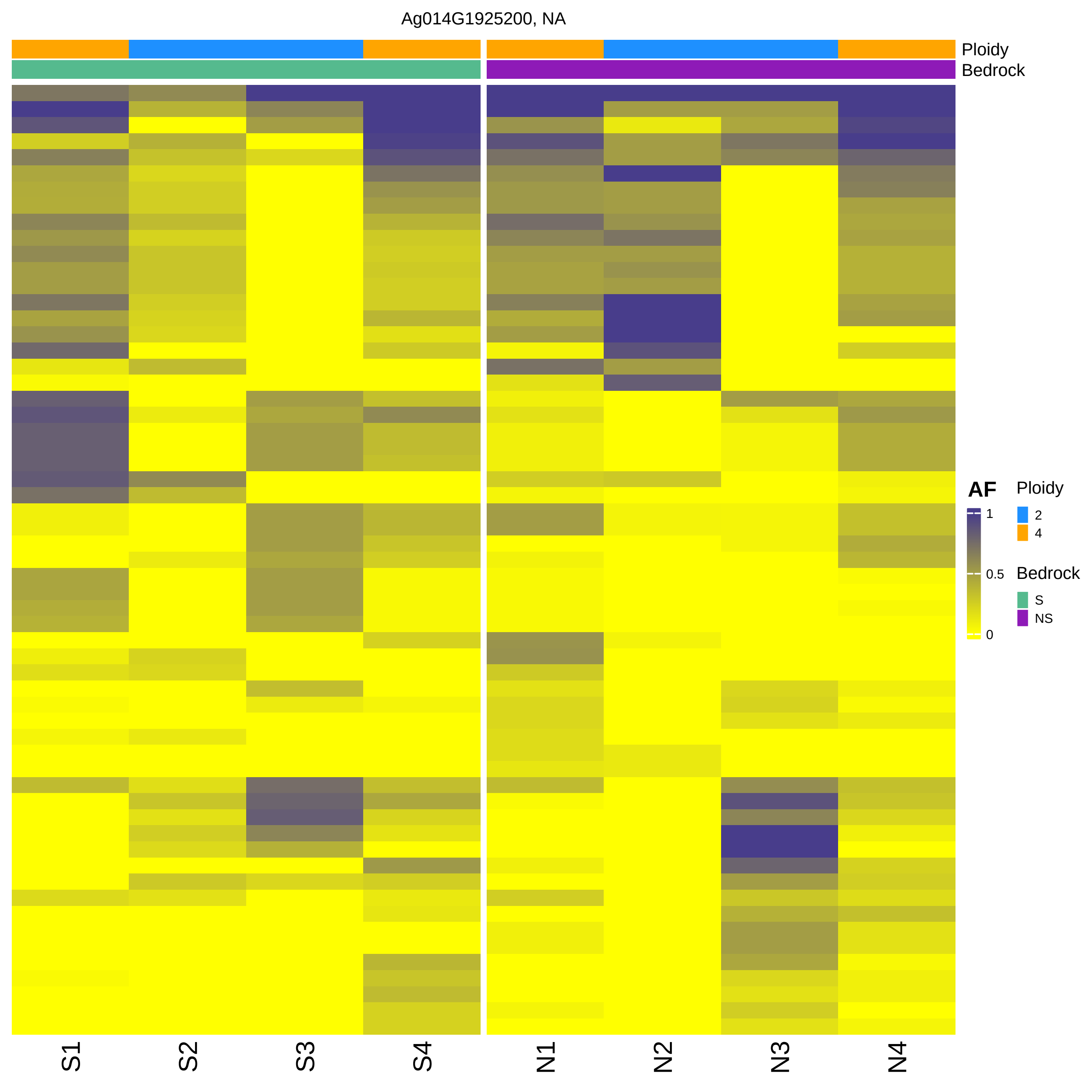

Ag014G1925200, NA
Ploidy
Bedrock
AF
1
0.5
0
Ploidy
2
4
Bedrock
S
NS
S1
S2
S3
S4
N1
N2
N3
N4

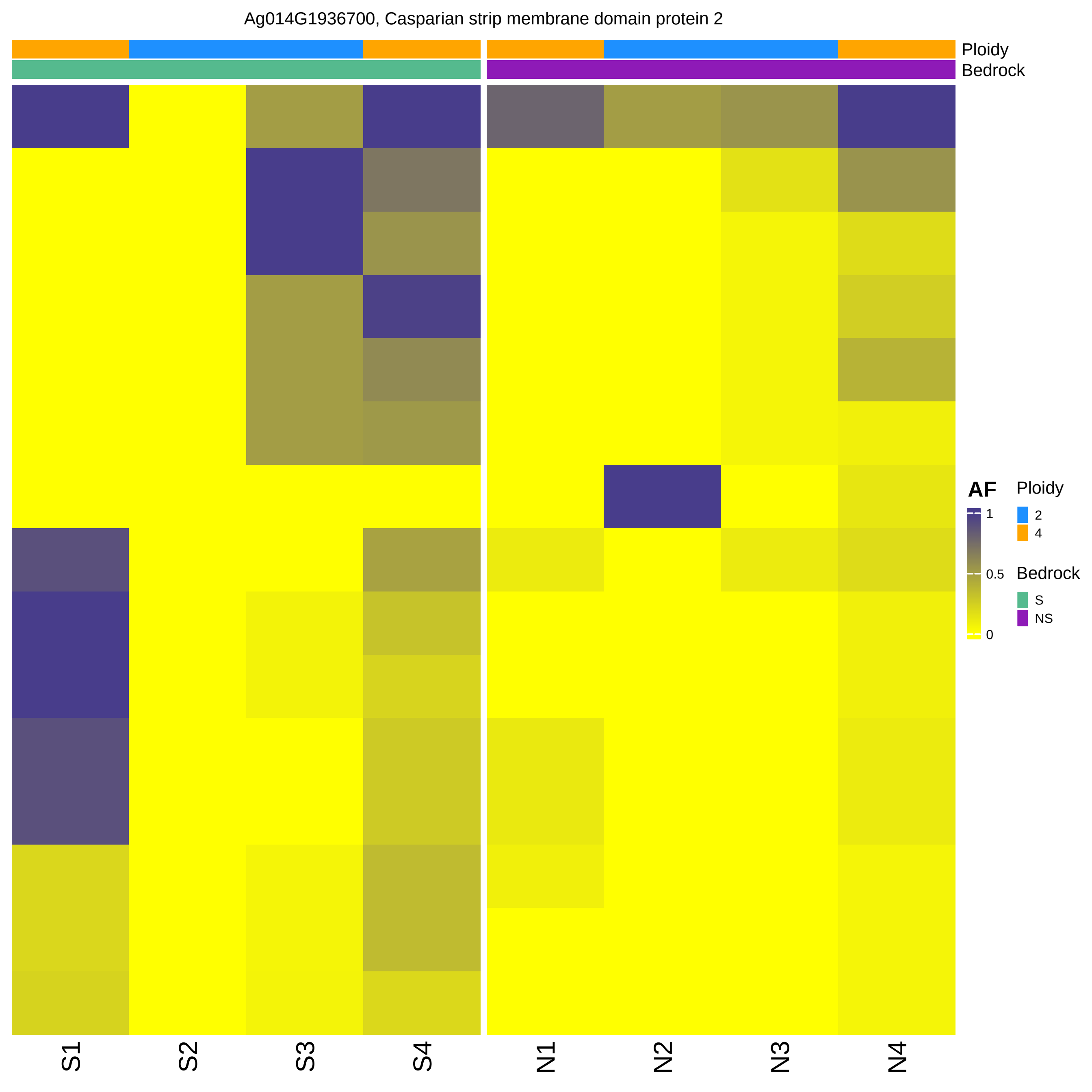
Ag014G1936700, Casparian strip membrane domain protein 2
Ploidy
Bedrock
AF
1
0.5
0
Ploidy
2
4
Bedrock
S
NS
S1
S2
S3
S4
N1
N2
N3
N4

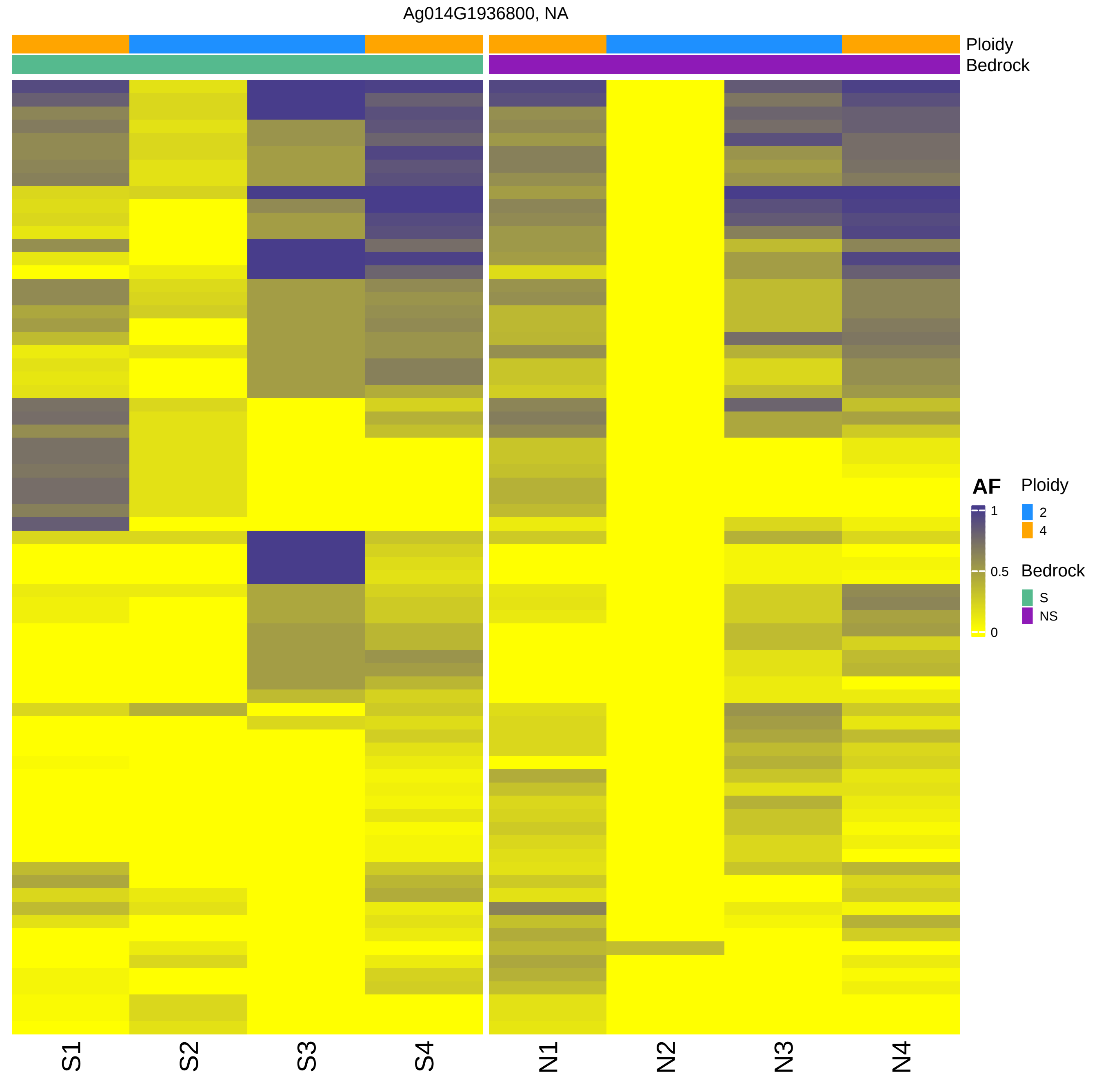

Ag014G1936800, NA
Ploidy
Bedrock
AF
1
0.5
0
Ploidy
2
4
Bedrock
S
NS
S1
S2
S3
S4
N1
N2
N3
N4

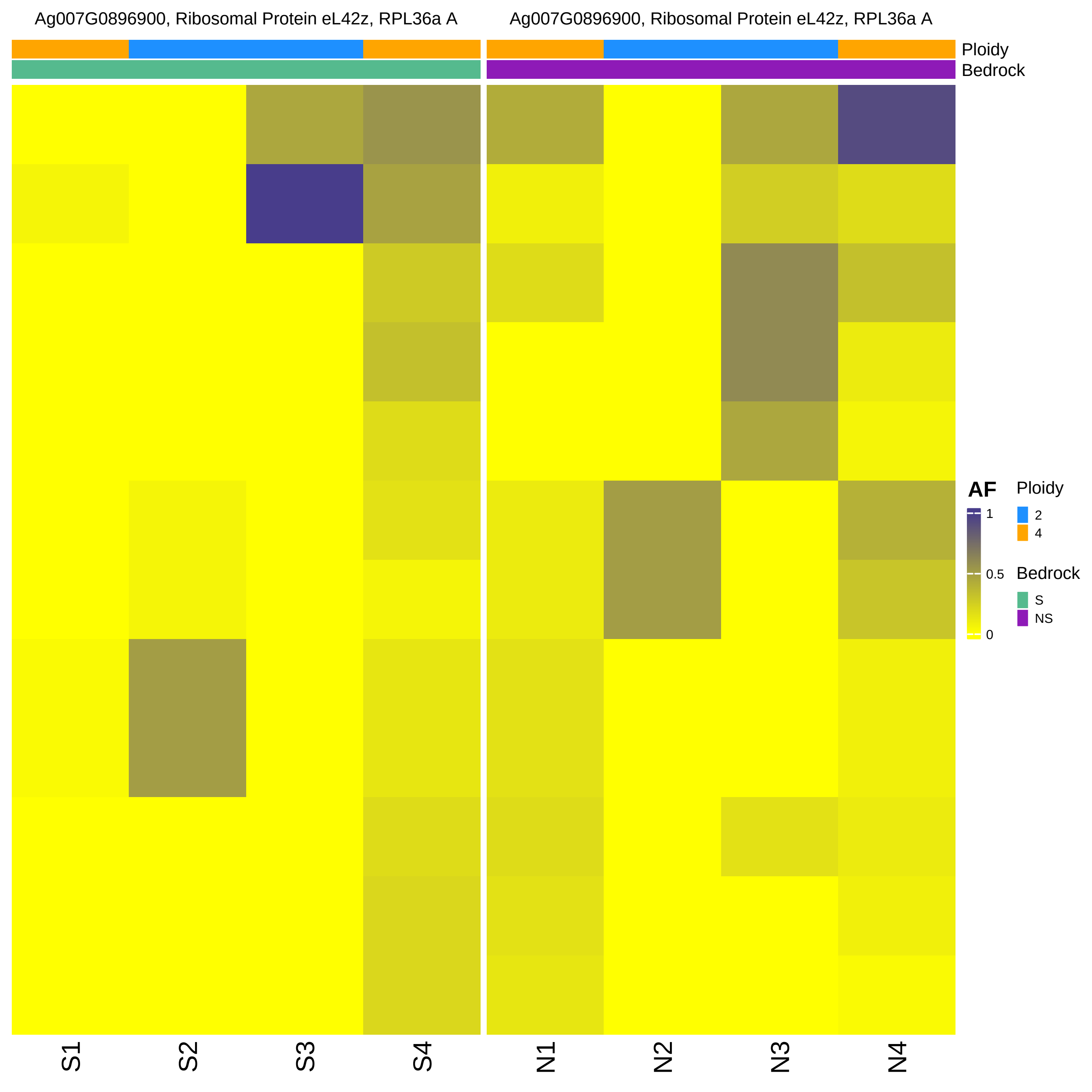

Ag007G0896900, Ribosomal Protein eL42z, RPL36a A
Ag007G0896900, Ribosomal Protein eL42z, RPL36a A
Ploidy
Bedrock
AF
1
0.5
0
Ploidy
2
4
Bedrock
S
NS
S1
S2
S3
S4
N1
N2
N3
N4

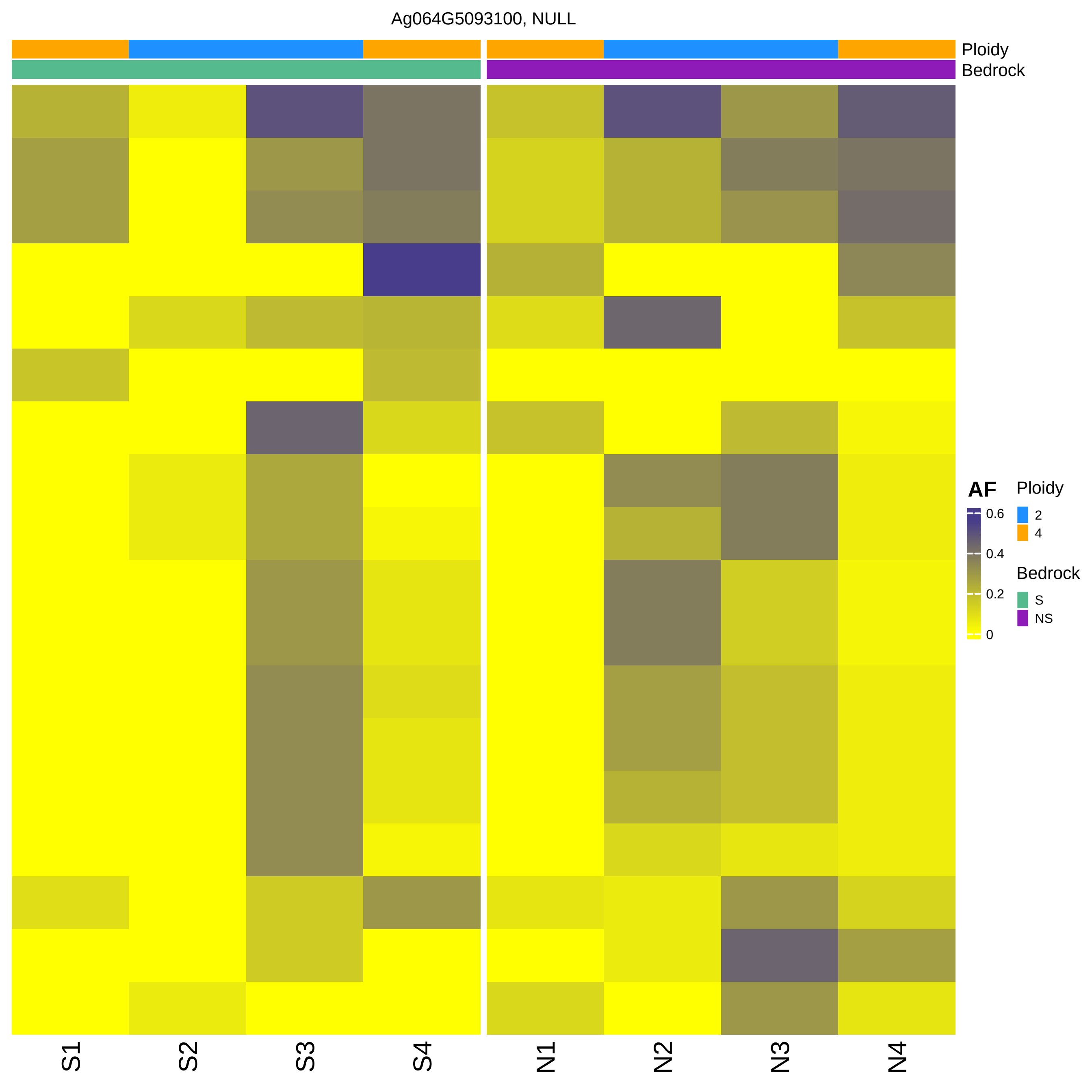

Ag064G5093100, NULL
Ploidy
Bedrock
AF
0.6
0.4
0.2
0
Ploidy
2
4
Bedrock
S
NS
S1
S2
S3
S4
N1
N2
N3
N4

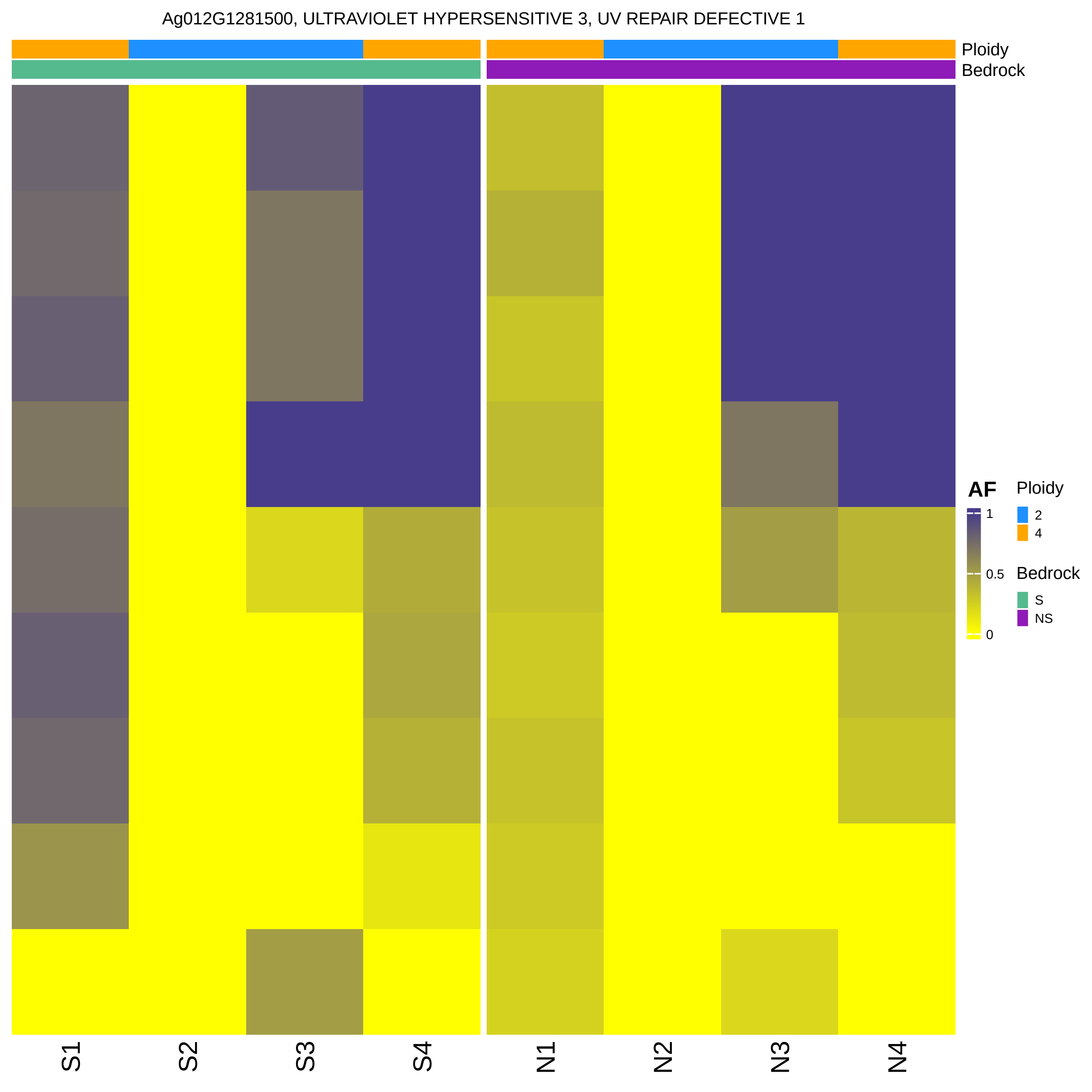

Ag012G1281500, ULTRAVIOLET HYPERSENSITIVE 3, UV REPAIR DEFECTIVE 1
Ploidy
Bedrock
AF
1
0.5
0
Ploidy
2
4
Bedrock
S
NS
S1
S2
S3
S4
N1
N2
N3
N4

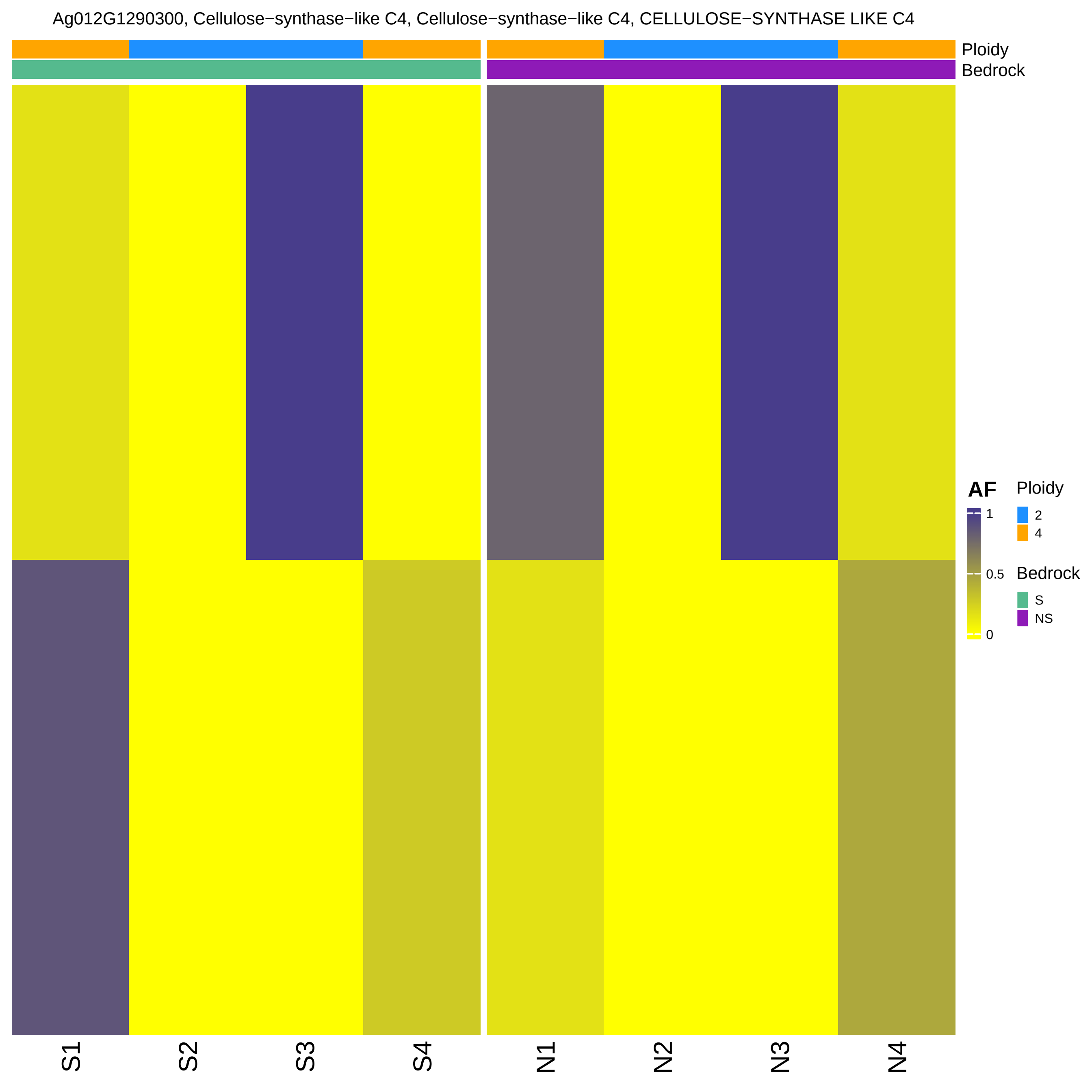
Ag012G1290300, Cellulose−synthase−like C4, Cellulose−synthase−like C4, CELLULOSE−SYNTHASE LIKE C4
Ploidy
Bedrock
AF
1
0.5
0
Ploidy
2
4
Bedrock
S
NS
S1
S2
S3
S4
N1
N2
N3
N4

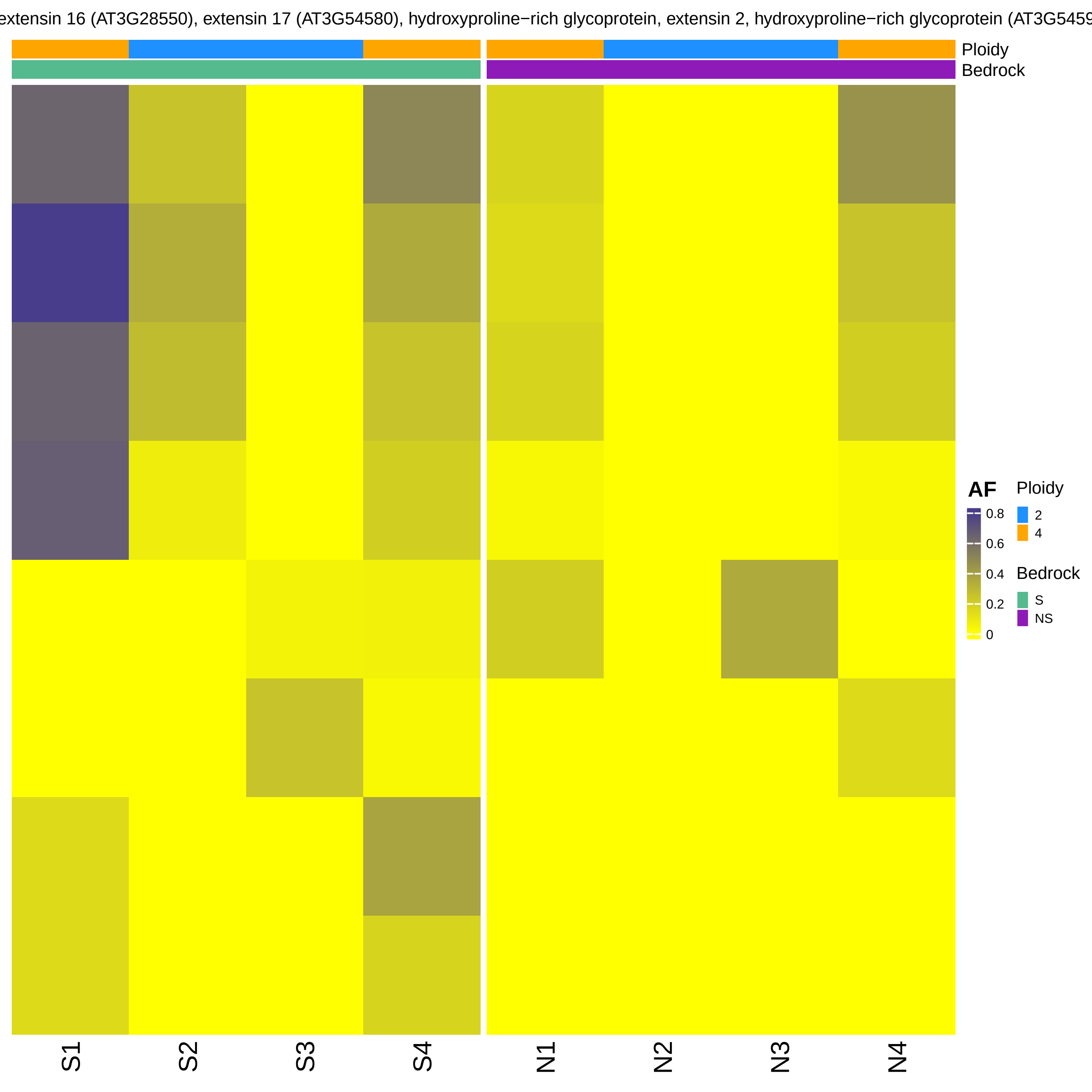
extensin 16 (AT3G28550), extensin 17 (AT3G54580), hydroxyproline−rich glycoprotein, extensin 2, hydroxyproline−rich glycoprotein (AT3G5459
Ploidy
Bedrock
AF
0.8
0.6
0.4
0.2
0
Ploidy
2
4
Bedrock
S
NS
S1
S2
S3
S4
N1
N2
N3
N4

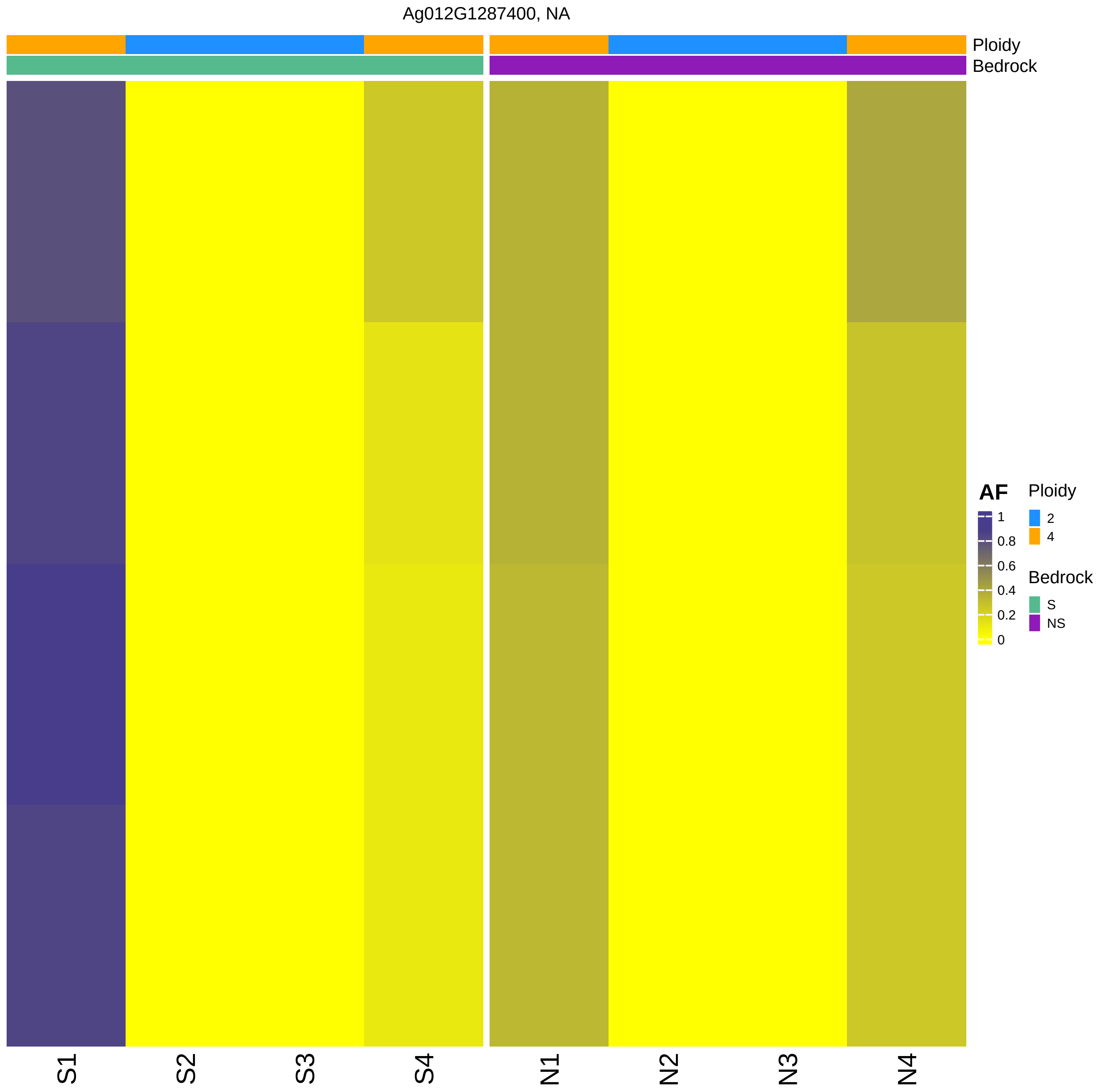
Ag012G1287400, NA
Ploidy
Bedrock
S1
S2
S3
S4
N1
N2
N3
N4
AF
1
0.8
0.6
0.4
0.2
0
Ploidy
2
4
Bedrock
S
NS

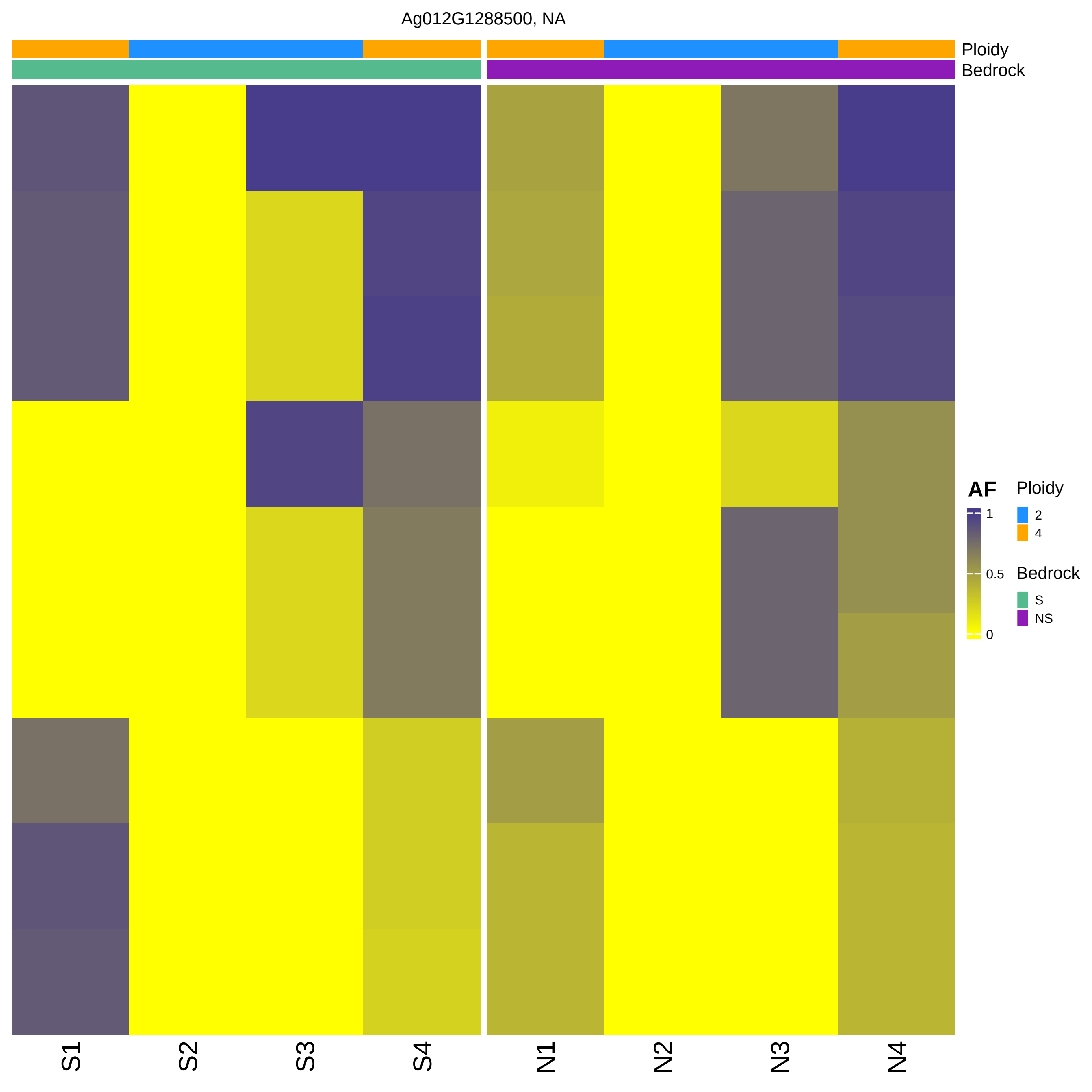

Ag012G1288500, NA
Ploidy
Bedrock
AF
1
0.5
0
Ploidy
2
4
Bedrock
S
NS
S1
S2
S3
S4
N1
N2
N3
N4

Ag012G1285700, Usually multiple acids move in and out Transporters 32

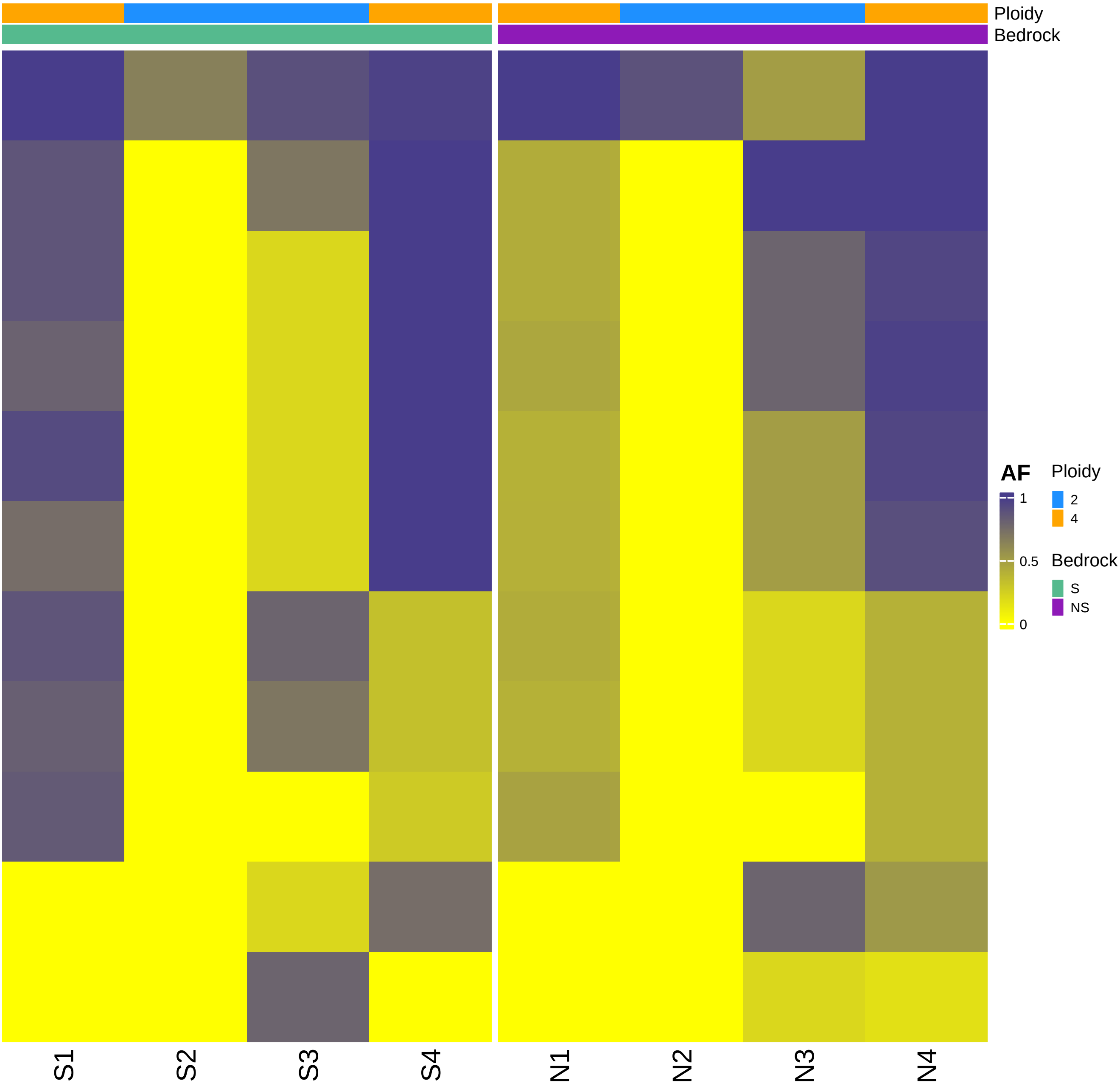

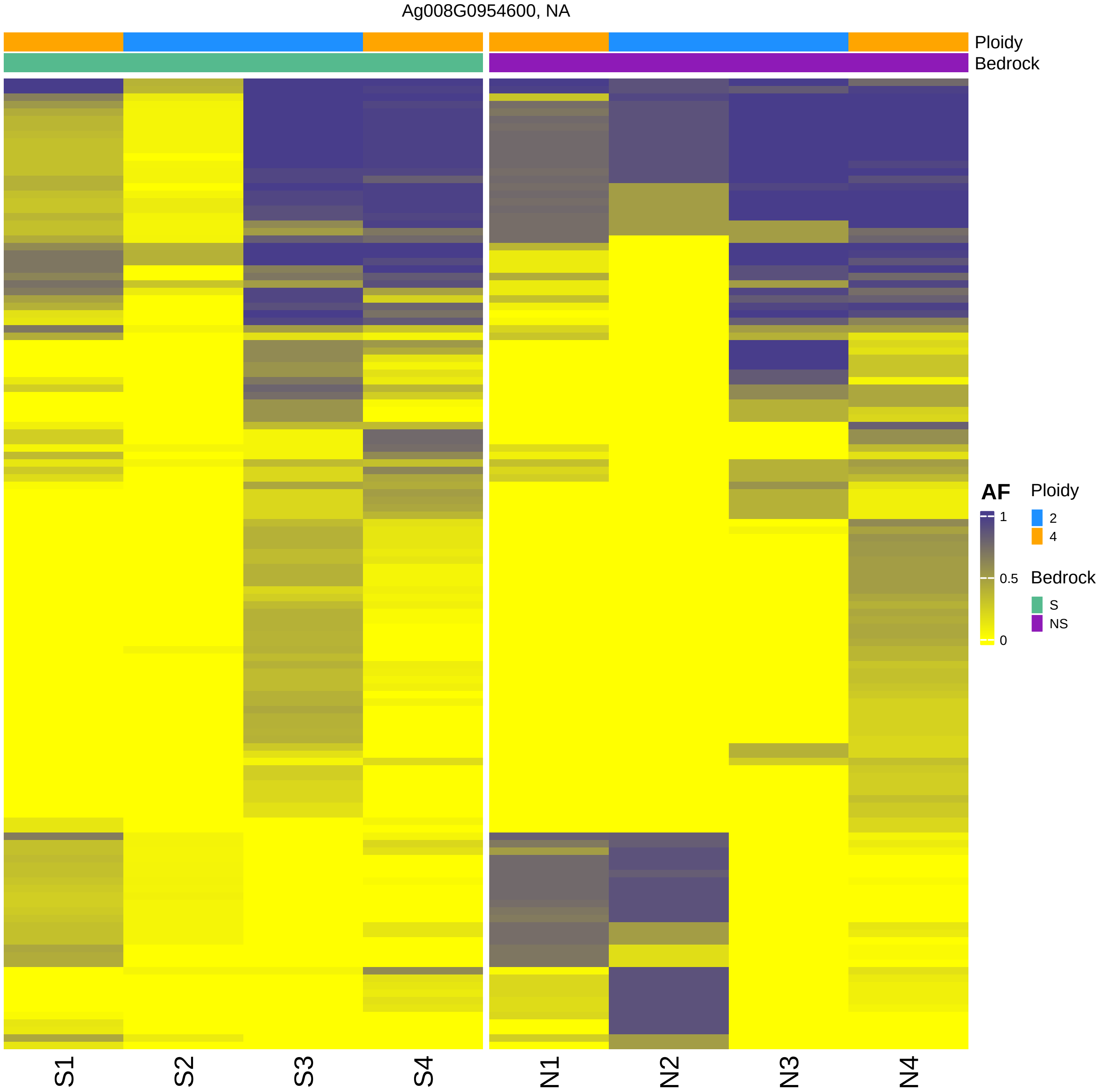
Ag008G0954600, NA
Ploidy
Bedrock
AF
1
0.5
0
Ploidy
2
4
Bedrock
S
NS
S1
S2
S3
S4
N1
N2
N3
N4

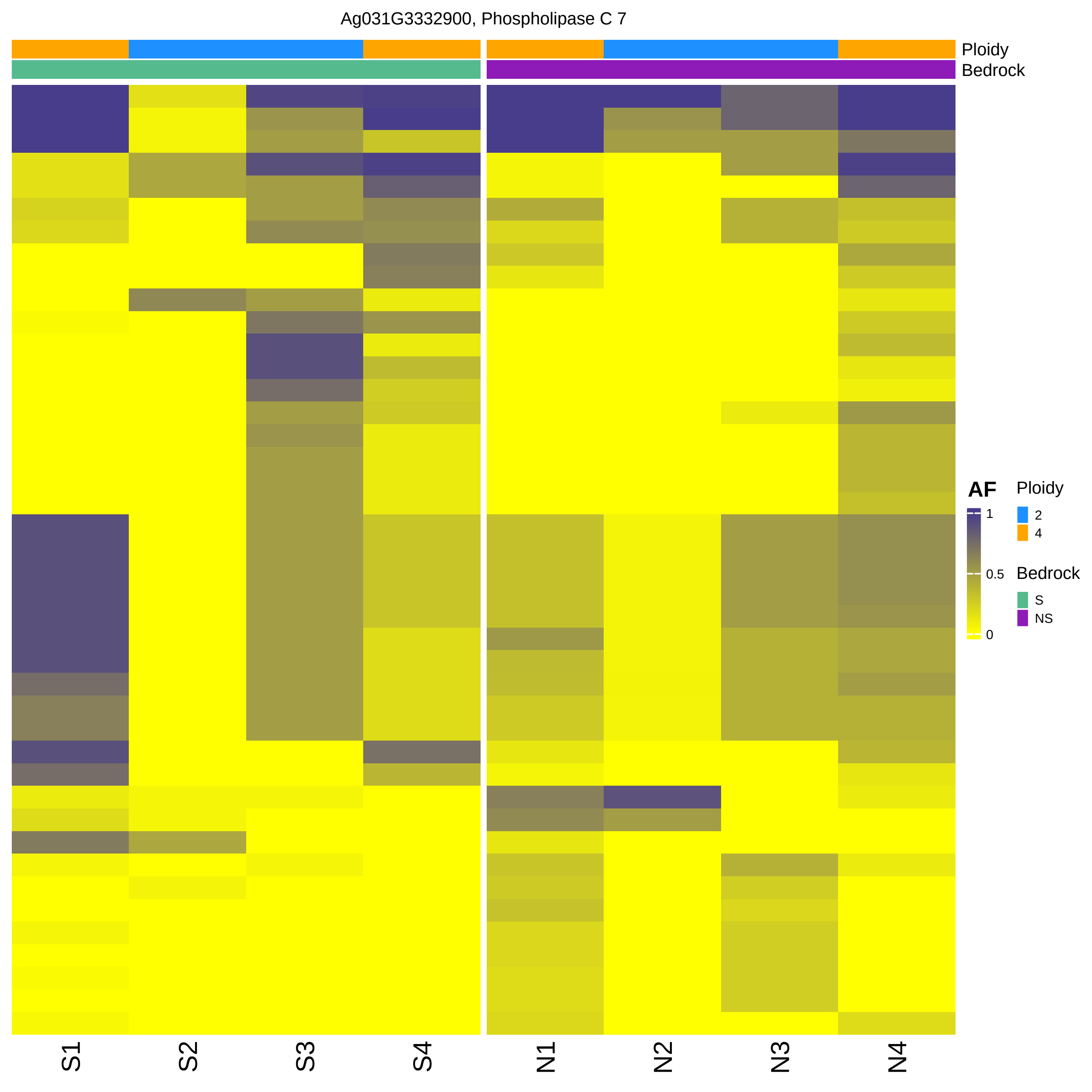

Ag031G3332900, Phospholipase C 7
Ploidy
Bedrock
AF
1
0.5
0
Ploidy
2
4
Bedrock
S
NS
S1
S2
S3
S4
N1
N2
N3
N4

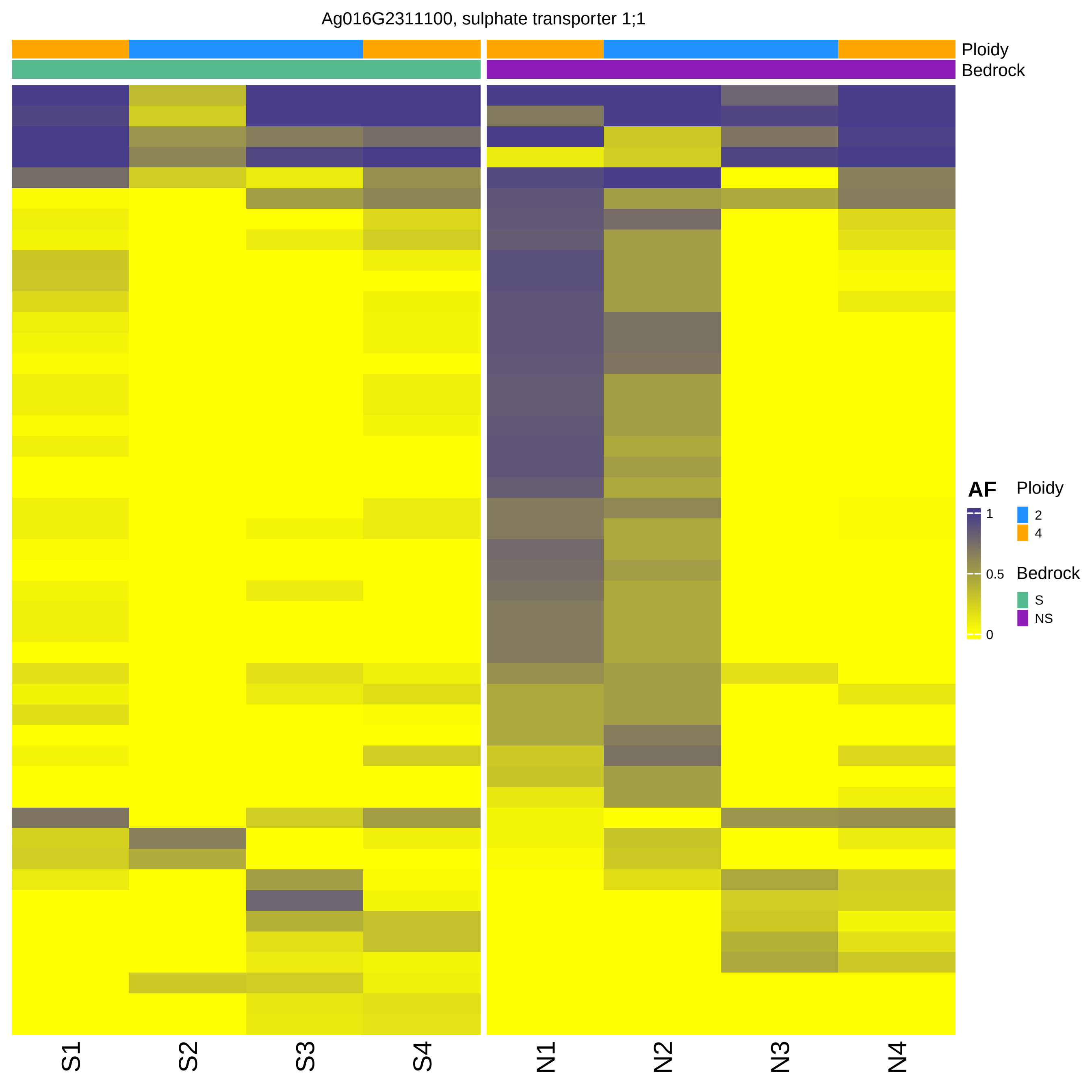

Ag016G2311100, sulphate transporter 1;1
Ploidy
Bedrock
AF
1
0.5
0
Ploidy
2
4
Bedrock
S
NS
S1
S2
S3
S4
N1
N2
N3
N4

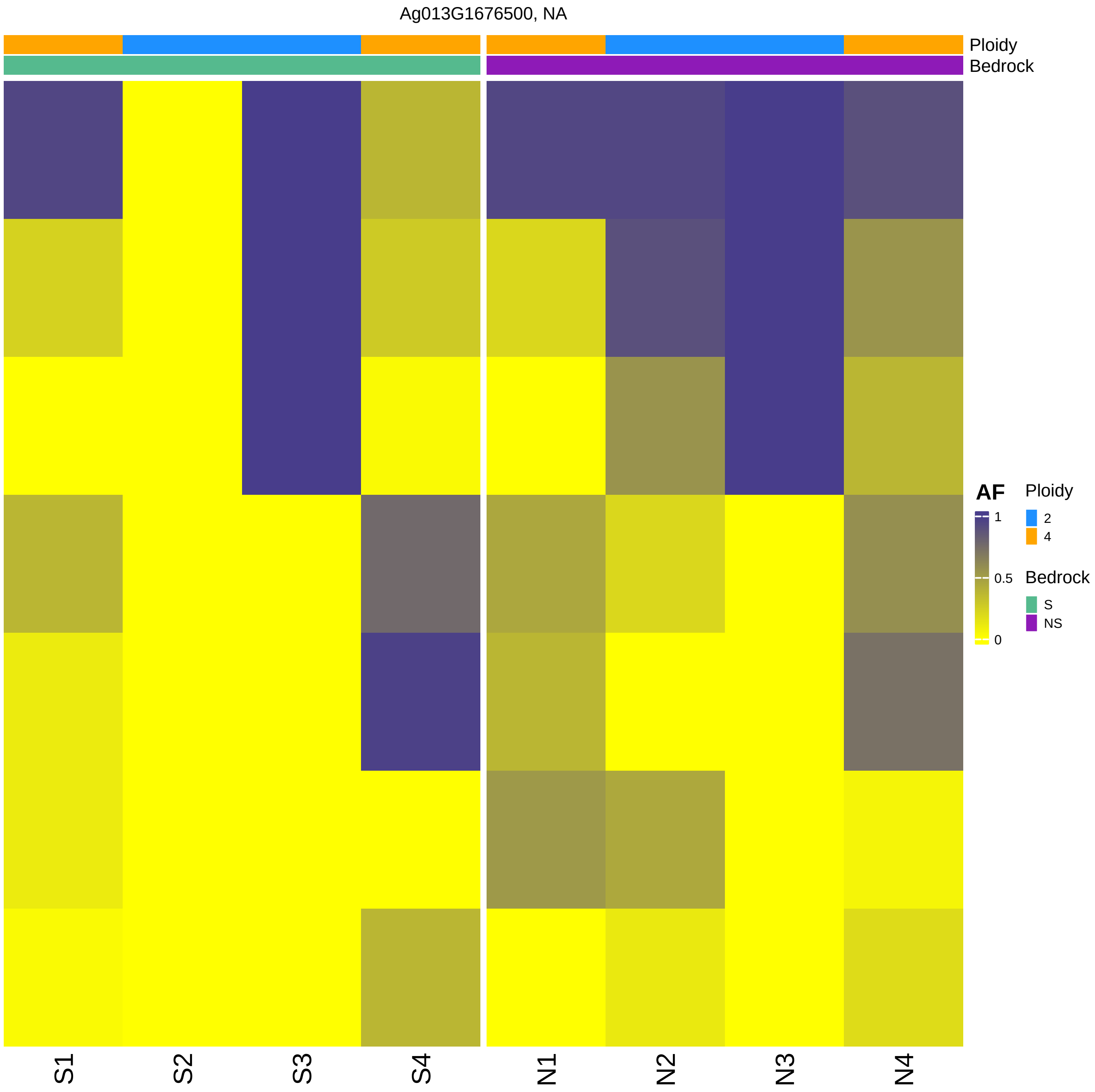

Ag013G1676500, NA
Ploidy
Bedrock
AF
1
0.5
0
Ploidy
2
4
Bedrock
S
NS
S1
S2
S3
S4
N1
N2
N3
N4

Ag007G0882200, NA

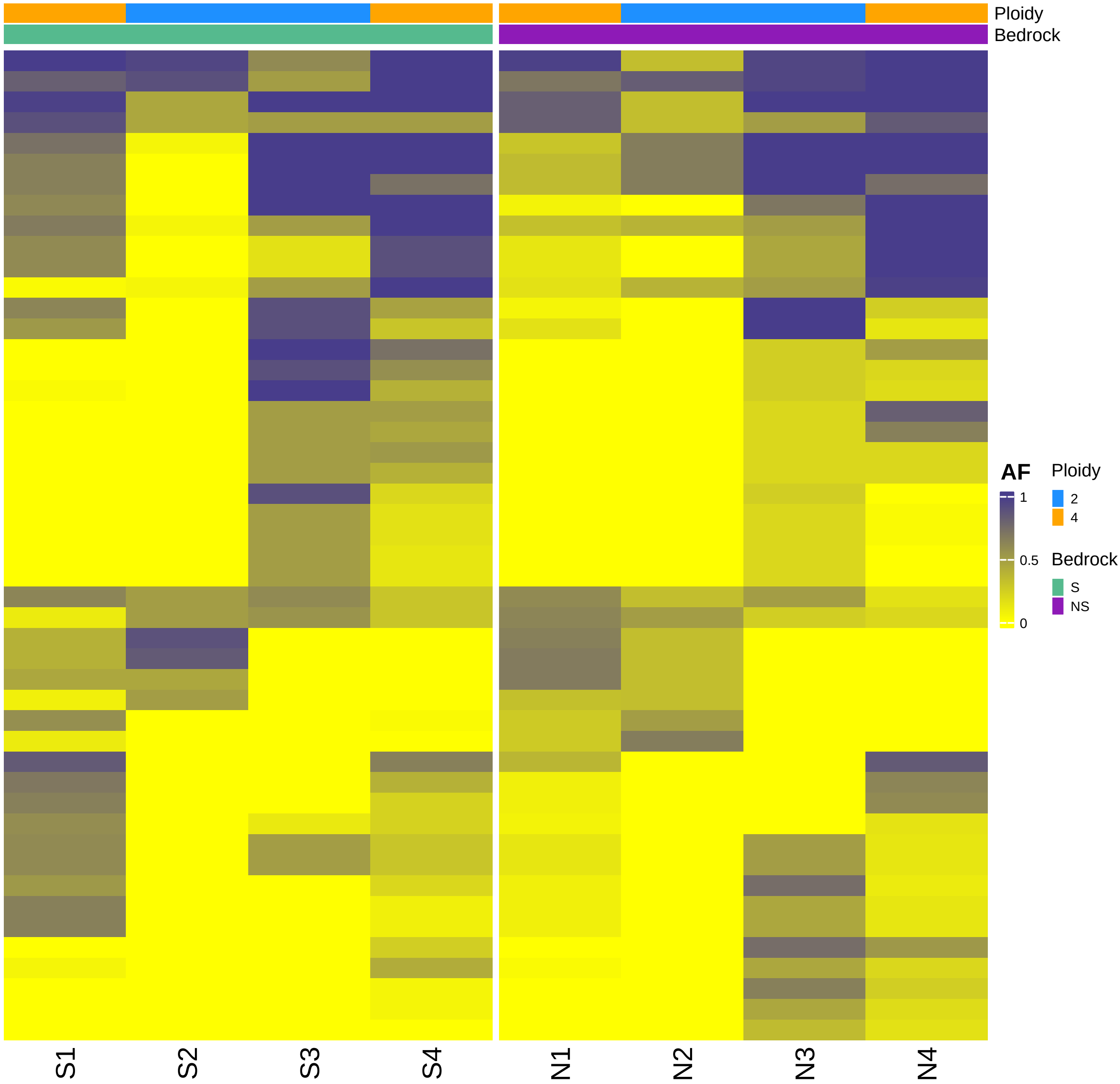

Ag007G0882400, ARABIDOPSIS THALIANA HIGH AFFINITY K+ TRANSPORTER 5, high affinity K+ transporter 5

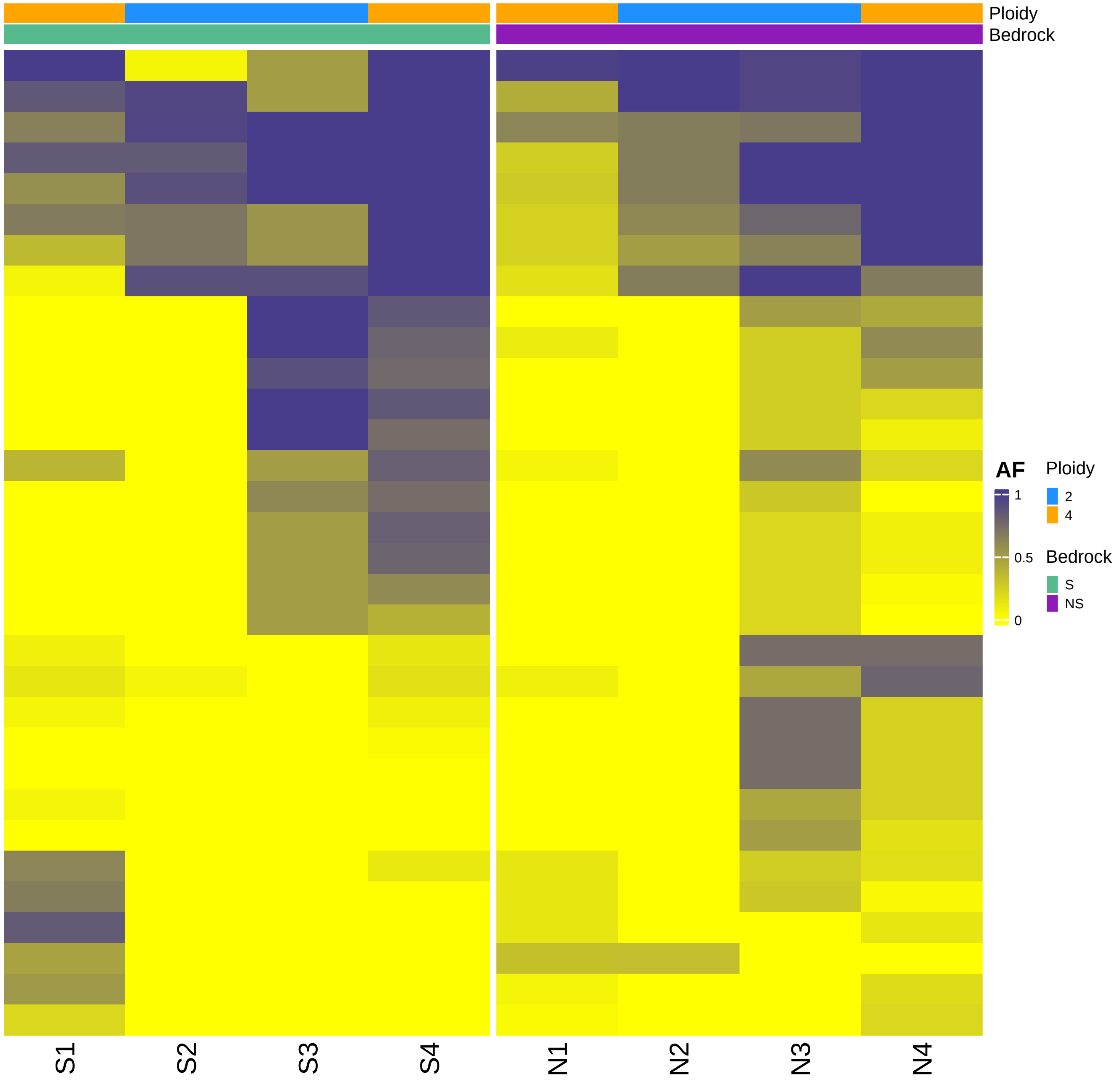

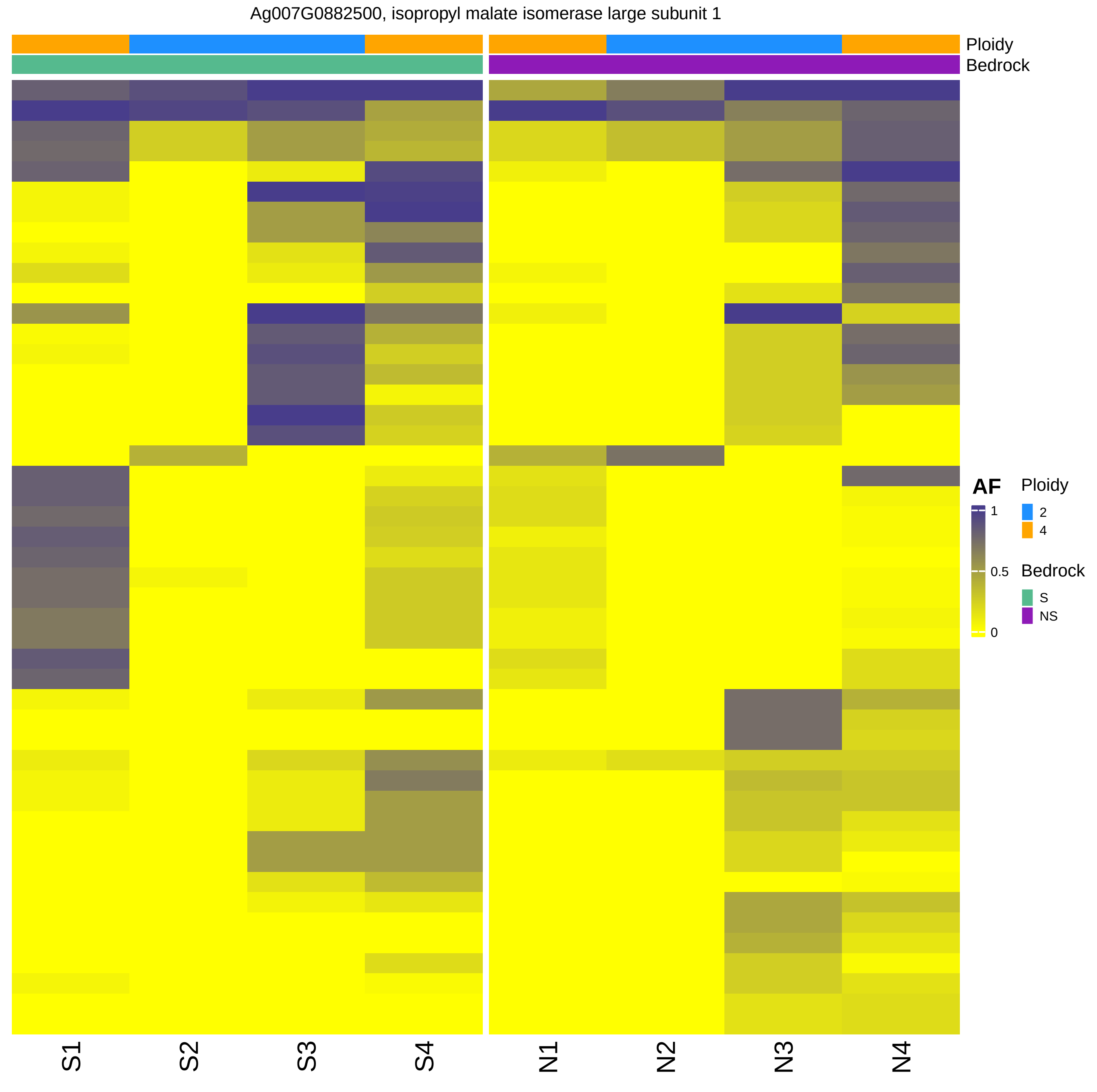

Ag007G0882500, isopropyl malate isomerase large subunit 1
Ploidy
Bedrock
AF
1
0.5
0
Ploidy
2
4
Bedrock
S
NS
S1
S2
S3
S4
N1
N2
N3
N4

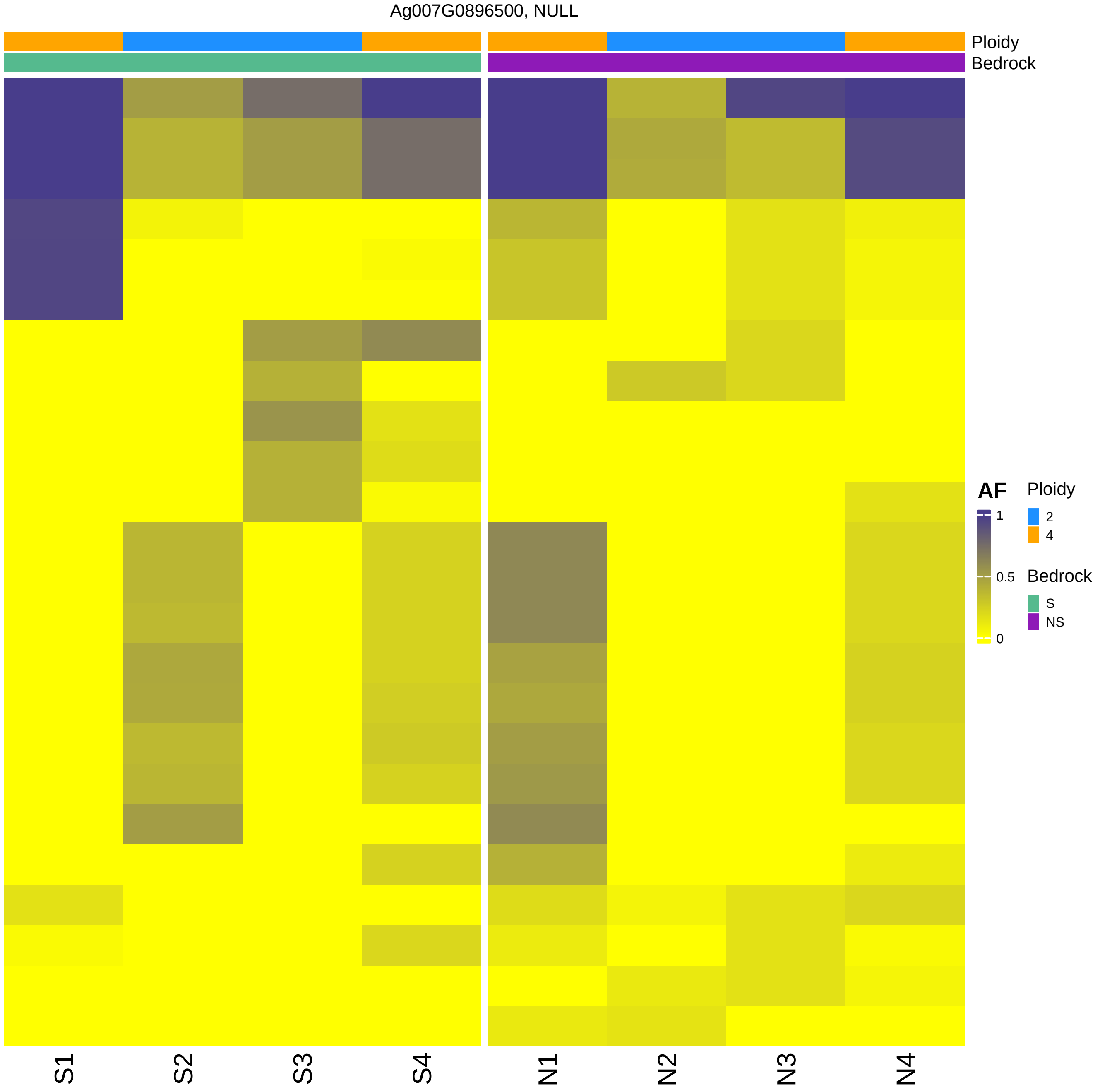
Ag007G0896500, NULL
Ploidy
Bedrock
AF
1
0.5
0
Ploidy
2
4
Bedrock
S
NS
S1
S2
S3
S4
N1
N2
N3
N4

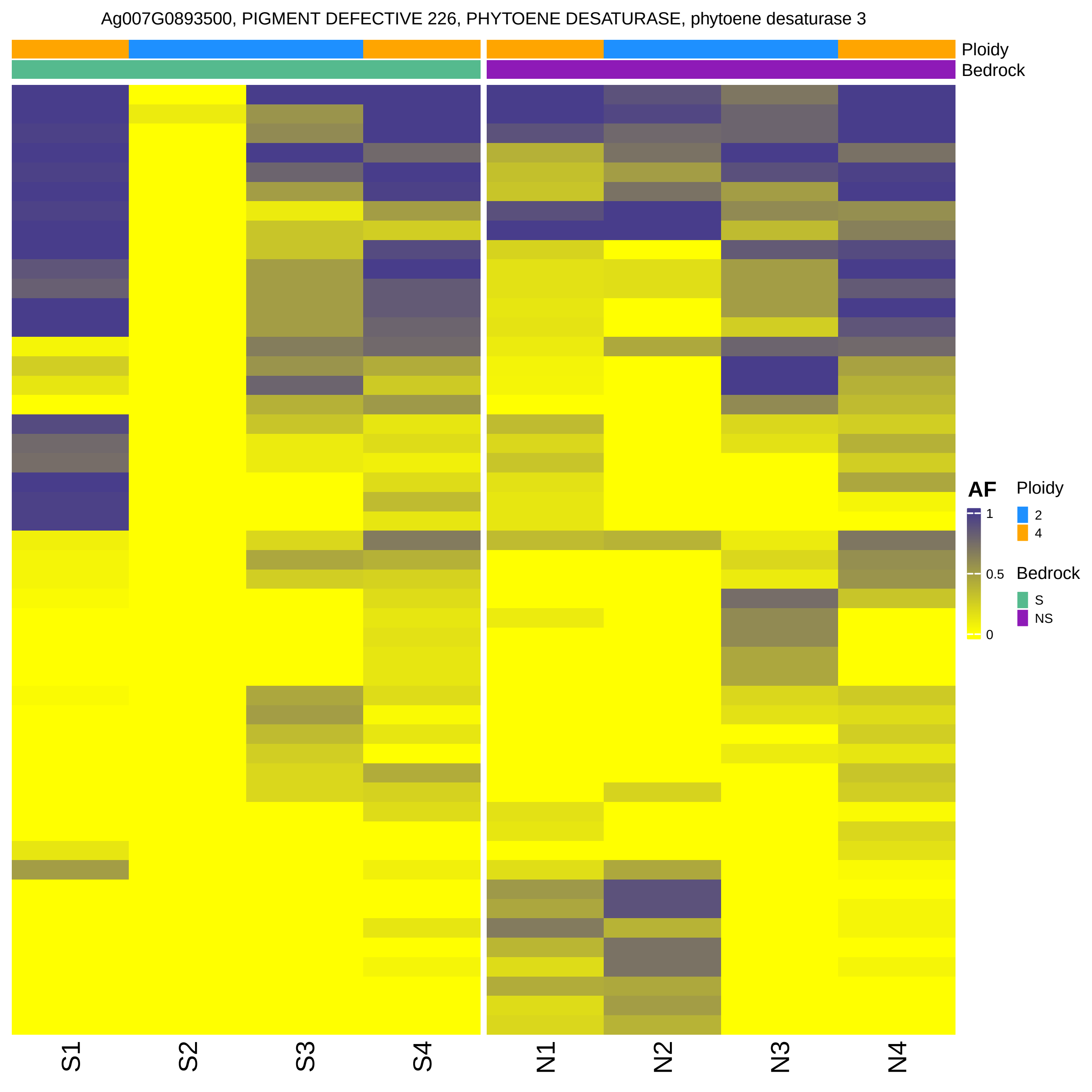
Ag007G0893500, PIGMENT DEFECTIVE 226, PHYTOENE DESATURASE, phytoene desaturase 3
Ploidy
Bedrock
AF
1
0.5
0
Ploidy
2
4
Bedrock
S
NS
S1
S2
S3
S4
N1
N2
N3
N4

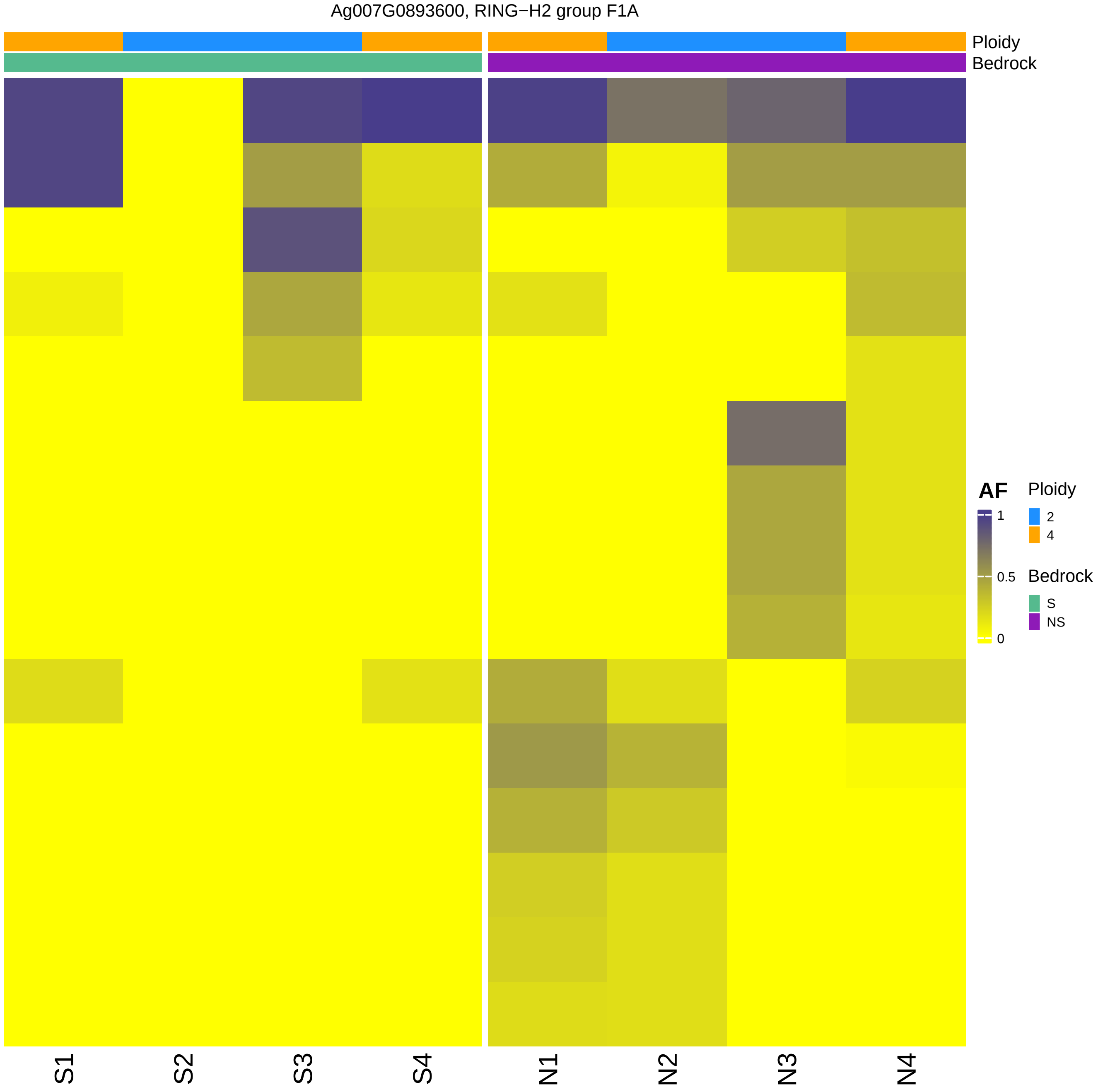

Ag007G0893600, RING−H2 group F1A
Ploidy
Bedrock
AF
1
0.5
0
Ploidy
2
4
Bedrock
S
NS
S1
S2
S3
S4
N1
N2
N3
N4

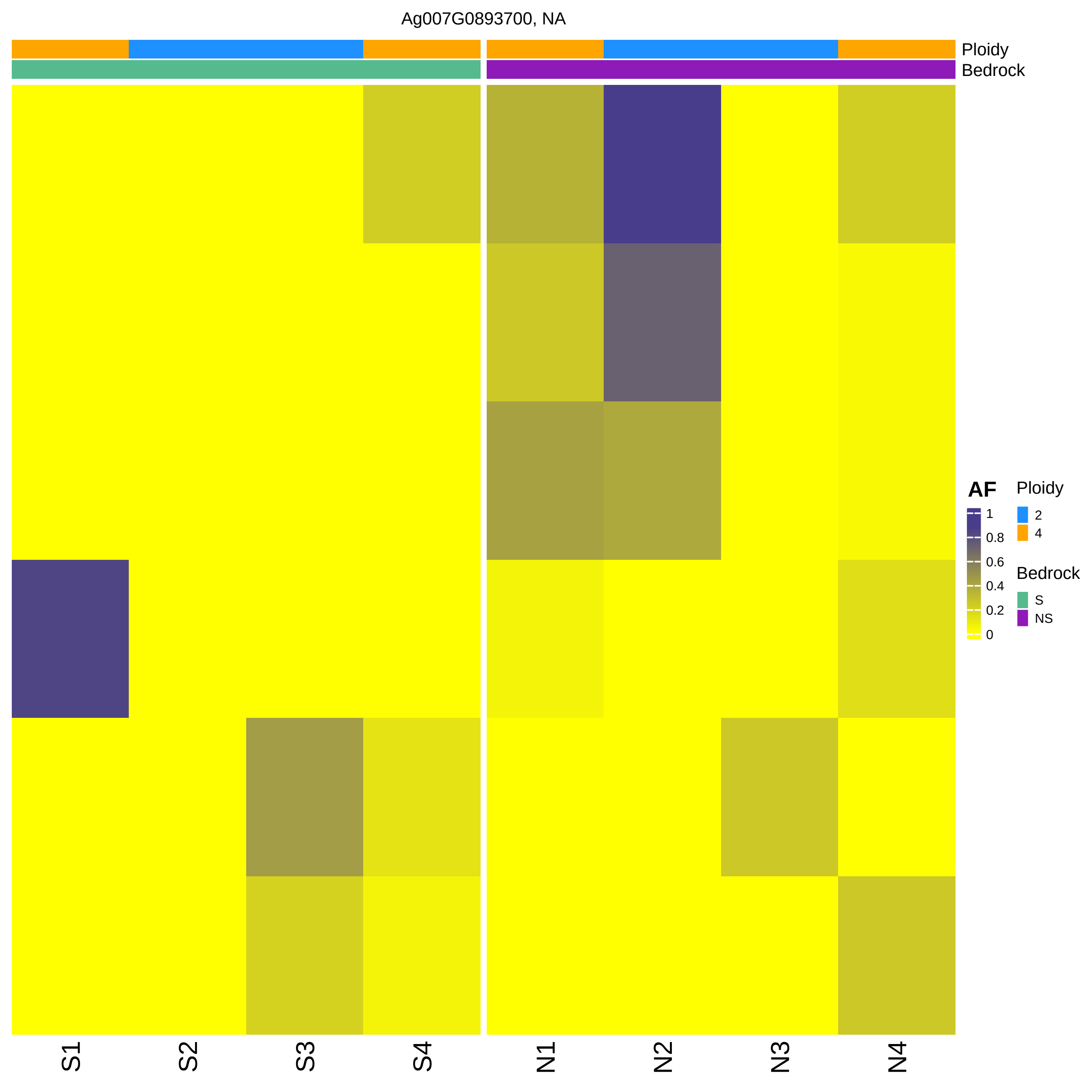
Ag007G0893700, NA
Ploidy
Bedrock
S1
S2
S3
S4
N1
N2
N3
N4
AF
1
0.8
0.6
0.4
0.2
0
Ploidy
2
4
Bedrock
S
NS

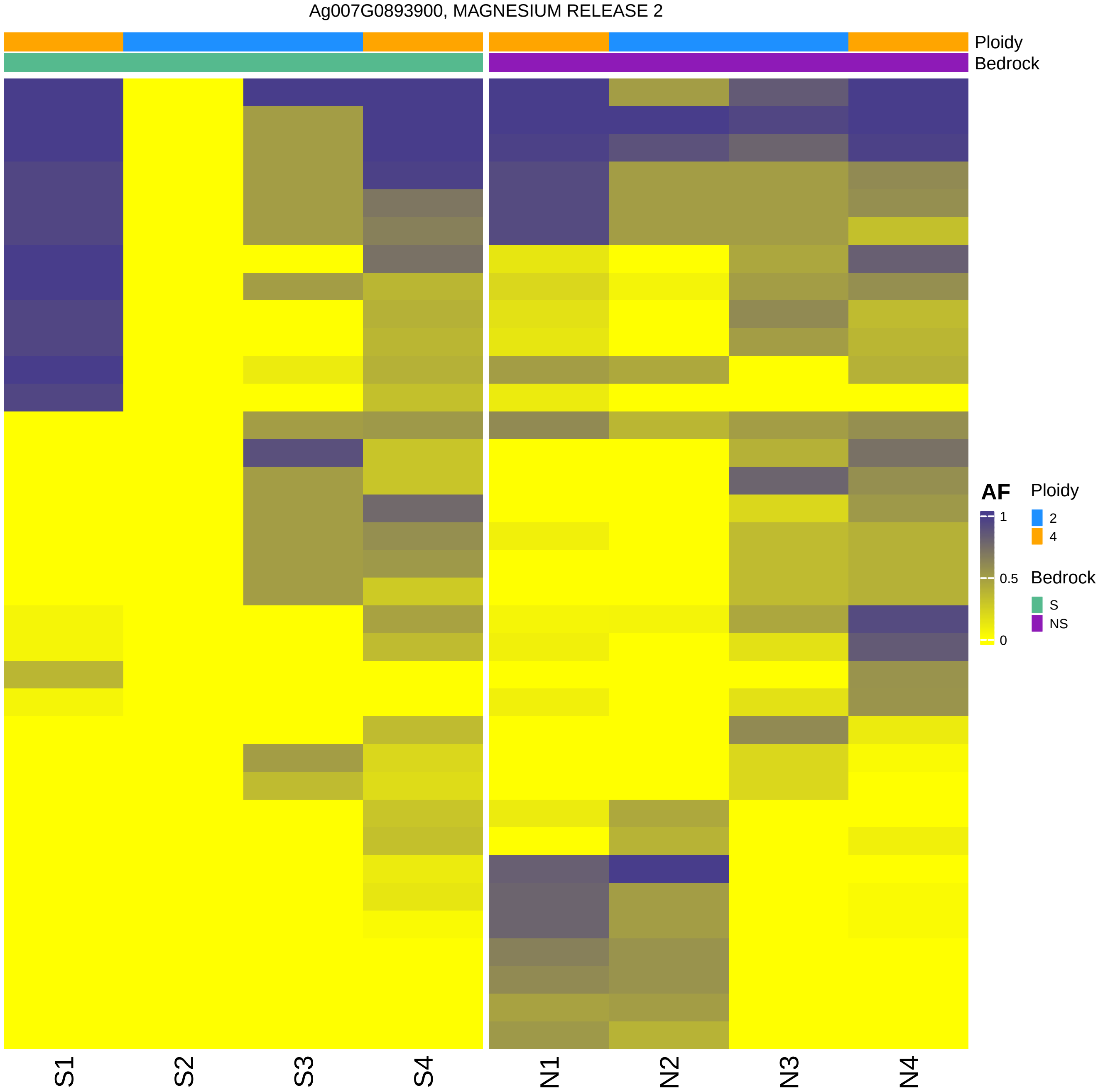

Ag007G0893900, MAGNESIUM RELEASE 2
Ploidy
Bedrock
AF
1
0.5
0
Ploidy
2
4
Bedrock
S
NS
S1
S2
S3
S4
N1
N2
N3
N4

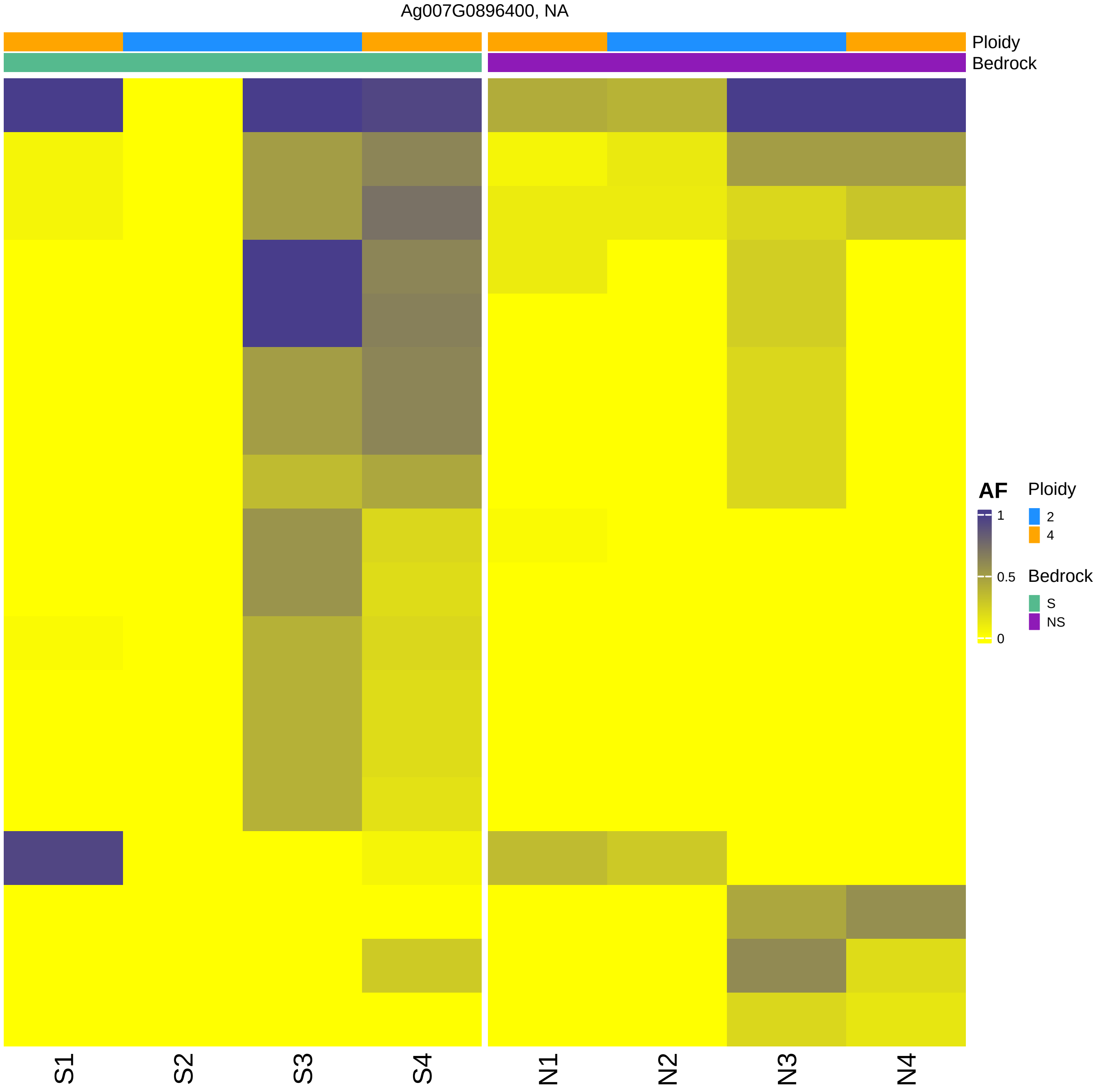
Ag007G0896400, NA
Ploidy
Bedrock
S1
S2
S3
S4
N1
N2
N3
N4
AF
1
0.5
0
Ploidy
2
4
Bedrock
S
NS

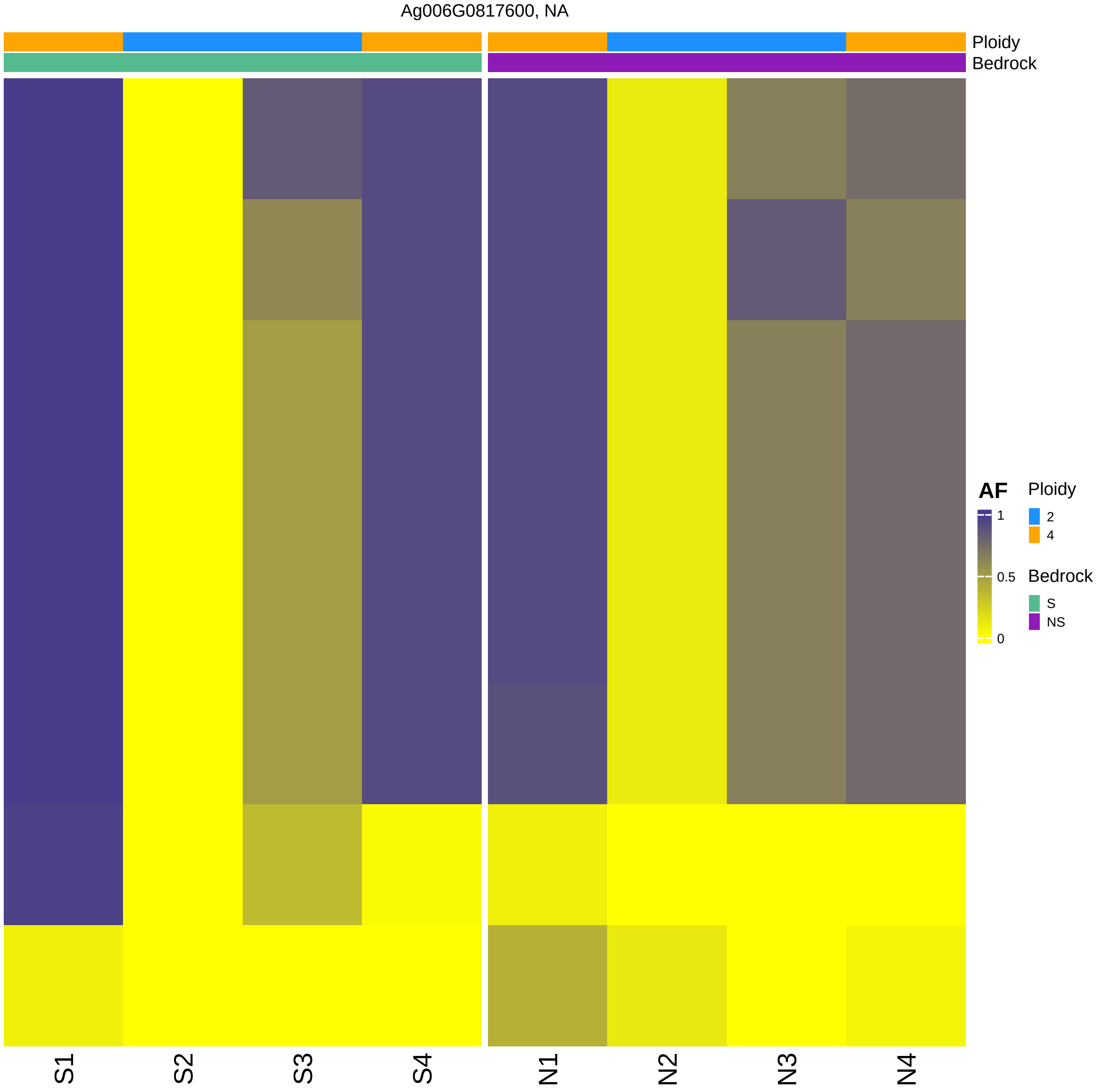
Ag006G0817600, NA
Ploidy
Bedrock
AF
1
0.5
0
Ploidy
2
4
Bedrock
S
NS
S1
S2
S3
S4
N1
N2
N3
N4

Ag011G1207800, MAGNESIUM RELEASE 6 (AT4G33700), MAGNESIUM RELEASE 7 (AT2G14520)

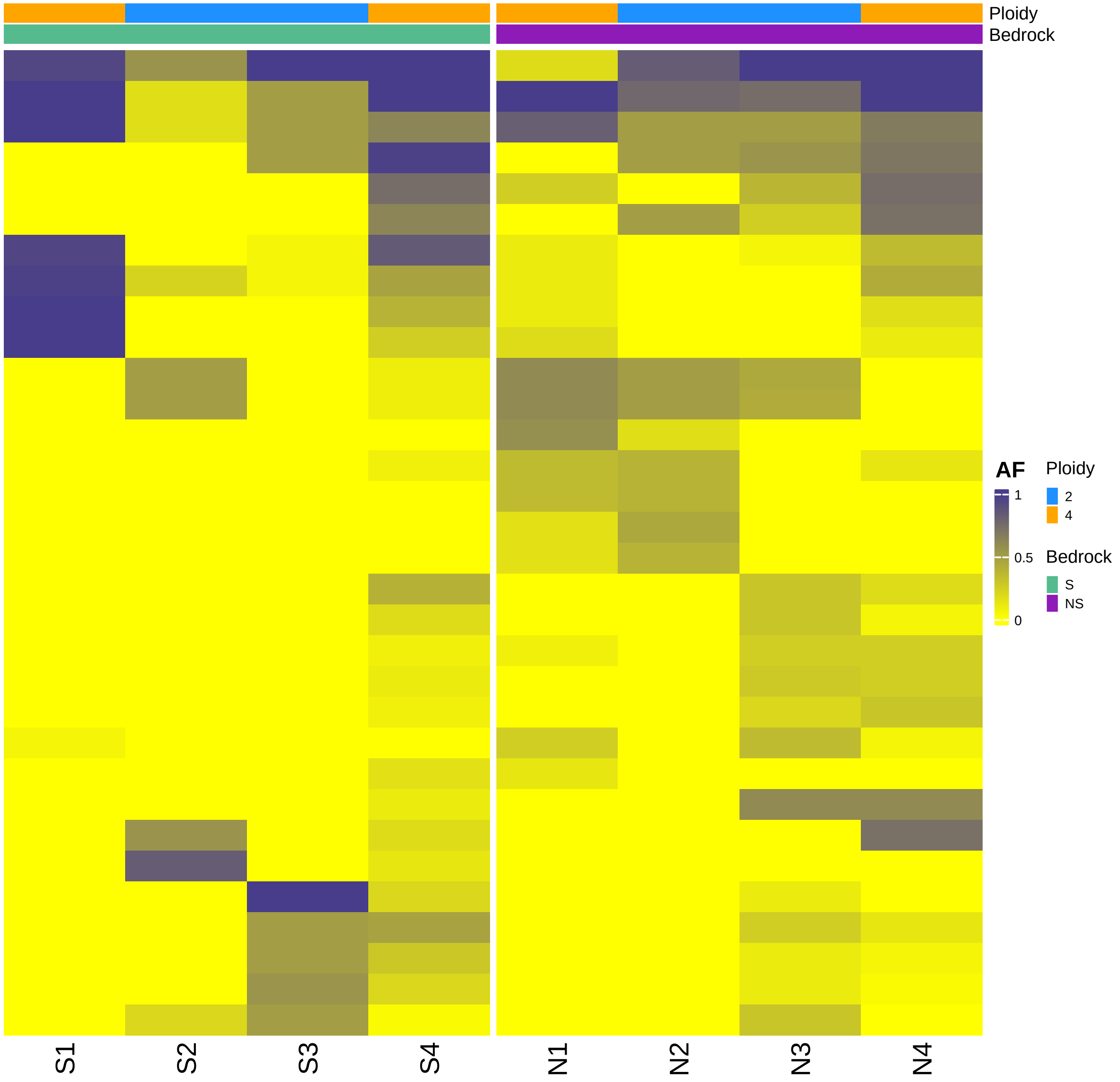

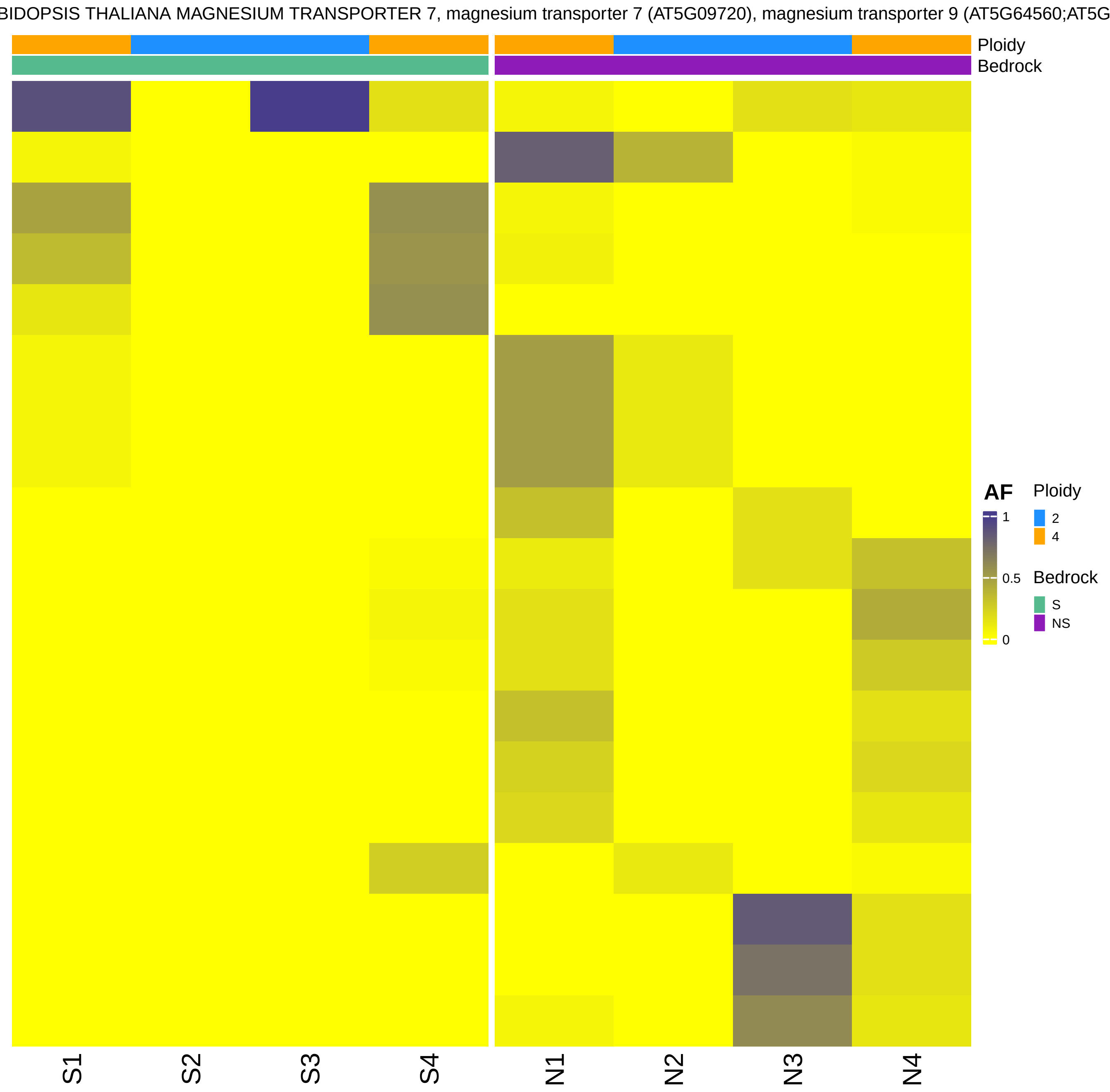

BIDOPSIS THALIANA MAGNESIUM TRANSPORTER 7, magnesium transporter 7 (AT5G09720), magnesium transporter 9 (AT5G64560;AT5G
Ploidy
Bedrock
AF
1
0.5
0
Ploidy
2
4
Bedrock
S
NS
S1
S2
S3
S4
N1
N2
N3
N4

04G0522600, ferric reduction oxidase 4 (AT5G23980), FERRIC REDUCTION OXIDASE 5, ferric reduction oxidase 5 (AT5G23990)

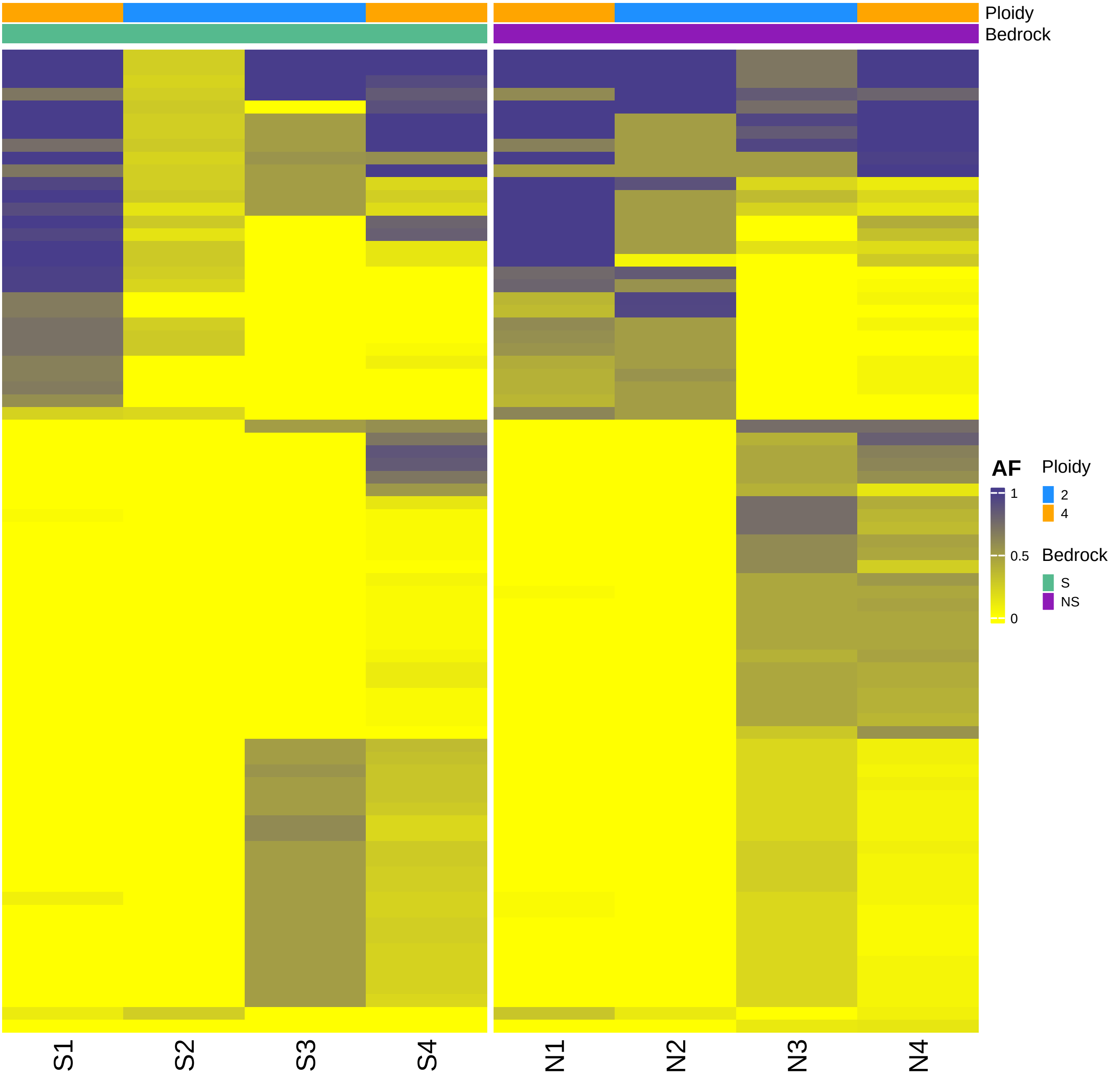

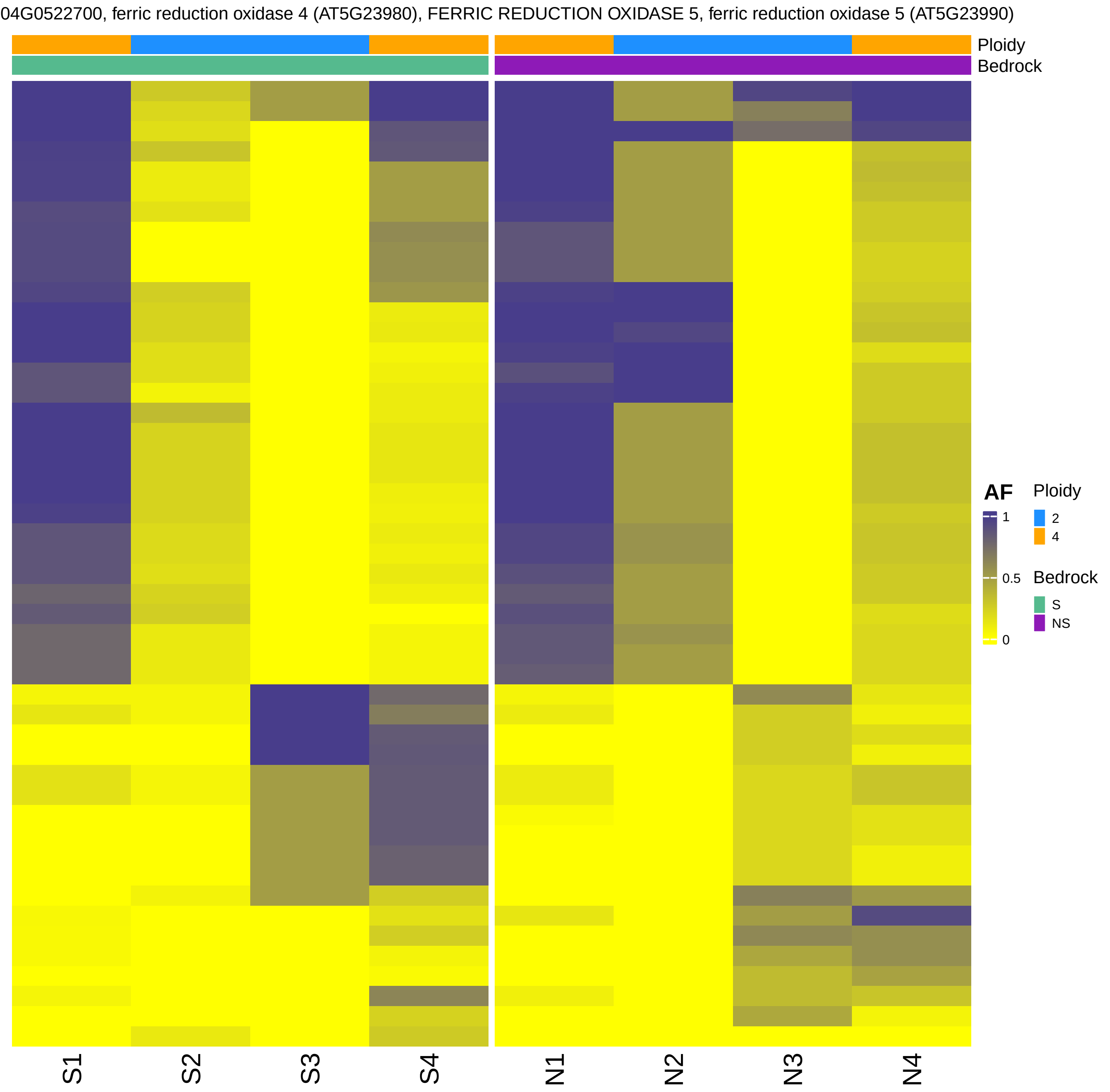

04G0522700, ferric reduction oxidase 4 (AT5G23980), FERRIC REDUCTION OXIDASE 5, ferric reduction oxidase 5 (AT5G23990)
Ploidy
Bedrock
S1
S2
S3
S4
N1
N2
N3
N4
AF
1
0.5
0
Ploidy
2
4
Bedrock
S
NS

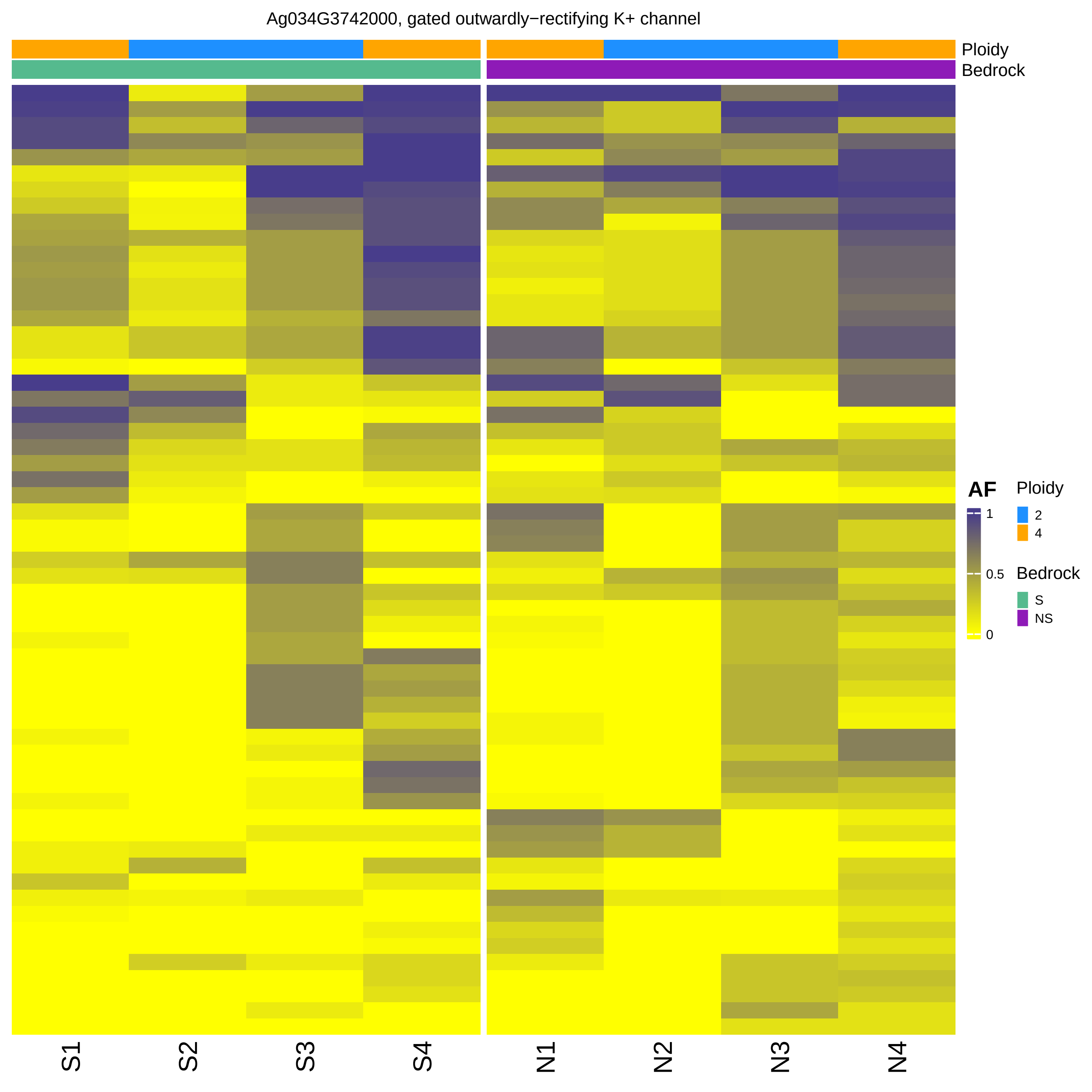

Ag034G3742000, gated outwardly−rectifying K+ channel
Ploidy
Bedrock
AF
1
0.5
0
Ploidy
2
4
Bedrock
S
NS
S1
S2
S3
S4
N1
N2
N3
N4
